# Functional characterization of the selective pan-allele anti-SIRPα antibody ADU-1805 that blocks the SIRPα–CD47 innate immune checkpoint

**DOI:** 10.1186/s40425-019-0772-0

**Published:** 2019-12-04

**Authors:** Erik Voets, Marc Paradé, David Lutje Hulsik, Sanne Spijkers, Wout Janssen, Joost Rens, Inge Reinieren-Beeren, Gilbert van den Tillaart, Sander van Duijnhoven, Lilian Driessen, Maurice Habraken, Peter van Zandvoort, Joost Kreijtz, Paul Vink, Andrea van Elsas, Hans van Eenennaam

**Affiliations:** 1 Aduro Biotech Europe B.V, Oss, The Netherlands; 2 Aduro Biotech, Inc., Berkeley, USA

**Keywords:** Cancer immunotherapy, SIRPα, CD47, Innate immune checkpoint, Myeloid cells

## Abstract

**Background:**

Accumulating preclinical data indicate that targeting the SIRPα/CD47 axis alone or in combination with existing targeted therapies or immune checkpoint inhibitors enhances tumor rejection. Although several CD47-targeting agents are currently in phase I clinical trials and demonstrate activity in combination therapy, high and frequent dosing was required and safety signals (acute anemia, thrombocytopenia) were recorded frequently as adverse events. Based on the restricted expression pattern of SIRPα we hypothesized that antibodies targeting SIRPα might avoid some of the concerns noted for CD47-targeting agents.

**Methods:**

SIRPα-targeting antibodies were generated and characterized for binding to human SIRPα alleles and blockade of the interaction with CD47. Functional activity was established in vitro using human macrophages or neutrophils co-cultured with human Burkitt’s lymphoma cell lines. The effect of SIRPα versus CD47 targeting on human T-cell activation was studied using an allogeneic mixed lymphocyte reaction and a *Staphylococcus* enterotoxin B-induced T-cell proliferation assay. Potential safety concerns of the selected SIRPα-targeting antibody were addressed in vitro using a hemagglutination assay and a whole blood cytokine release assay, and in vivo in a single-dose toxicity study in cynomolgus monkeys.

**Results:**

The humanized monoclonal IgG2 antibody ADU-1805 binds to all known human SIRPα alleles, showing minimal binding to SIRPβ1, while cross-reacting with SIRPγ, and potently blocking the interaction of SIRPα with CD47. Reduced FcγR binding proved critical to retaining its function towards phagocyte activation. In vitro characterization demonstrated that ADU-1805 promotes macrophage phagocytosis, with similar potency to anti-CD47 antibodies, and enhances neutrophil trogocytosis. Unlike CD47-targeting agents, ADU-1805 does not interfere with T-cell activation and is not expected to require frequent and extensive dosing due to the restricted expression of SIRPα to cells of the myeloid lineage. ADU-1805 is cross-reactive to cynomolgus monkey SIRPα and upon single-dose intravenous administration in these non-human primates (NHPs) did not show any signs of anemia, thrombocytopenia or other toxicities.

**Conclusions:**

Blocking the SIRPα-CD47 interaction via SIRPα, while similarly efficacious in vitro, differentiates ADU-1805 from CD47-targeting agents with respect to safety and absence of inhibition of T-cell activation. The data presented herein support further advancement of ADU-1805 towards clinical development.

## Background

Analogous to the well-established T-cell immune checkpoints (i.e. PD-1, CTLA-4), signal-regulatory protein α (SIRPα) is regarded as an innate immune checkpoint expressed on dendritic cells, macrophages, monocytes and neutrophils [1]. SIRPα is an inhibitory receptor and member of the so-called paired immune receptor family and has several ligands including the surfactant proteins (e.g. Sp-A and Sp-D) [2], and CD47 [3]. CD47 serves as a “self molecule” signal with its best-characterized functions in the homeostasis of complement- or Ig-opsonized red blood cells (RBCs) and platelets. Binding of CD47 to SIRPα inhibits phagocytosis of these cells by macrophages thereby preventing their homeostatic clearance [4, 5].

The overexpression of CD47 on numerous human cancers [6–11] suggested that tumor cells may evade phagocytosis and clearance by upregulating CD47 expression. Targeting of the SIRPα/CD47 axis in the context of cancer using an anti-CD47 blocking antibody enhanced phagocytosis of acute myeloid leukemia (AML) cells [6]. In addition, targeting the SIRPα/CD47 axis enhances tumor growth inhibition by existing tumor-targeting monoclonal antibody (mAb) therapies (e.g. rituximab, trastuzumab, alemtuzumab, daratumumab and cetuximab) [8, 12–14] and synergizes with other treatments including chemotherapy [15], radiotherapy [16], targeted therapy using small-molecule drugs [17] as well as immunotherapeutic agents blocking the PD-1/PD-L1 axis [18, 19].

Numerous agents blocking the SIRPα-CD47 innate immune checkpoint have been developed thus far including anti-CD47 and anti-SIRPα antibodies, and soluble SIRPαFc, of which several are currently being evaluated in clinical trials. Of these, Hu5F9-G4, TTI-621 and ALX148 are furthest in development and have shown encouraging clinical data either alone or in combination with other agents [14, 20, 21]. Nevertheless, the systemic use of CD47-targeting agents is thought to be hampered by the broad expression of CD47, which is manifested by severe depletion of RBCs and platelets, leading to acute anemia and thrombocytopenia in treated patients [20, 22] and requiring substantial amounts of agent to block CD47 on all immune cells (i.e. the “antigen sink”). Furthermore, CD47 is also a receptor for thrombospondin-1 (TSP1) [23] and blocking this interaction with anti-CD47 mAbs may have additional undesirable effects [24].

**Fig. 1:**
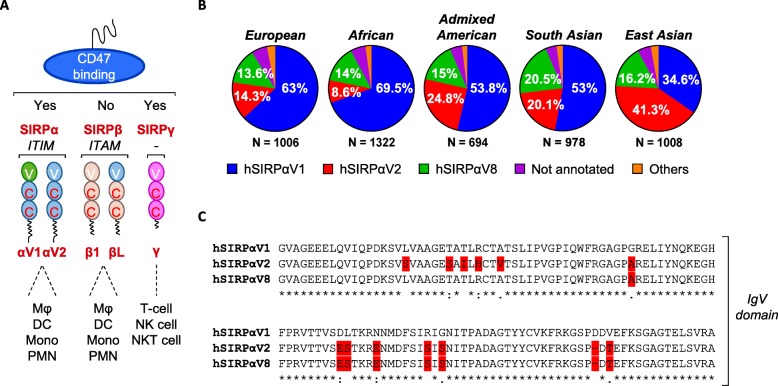
SIRPαV1, SIRPαV2, and SIRPαV8 are the main SIRPα variants in human. **a** The SIRP family of paired receptors comprises inhibitory (SIRPα), activating (SIRPβ) and non-signaling (SIRPγ) members. Mϕ, macrophage; DC, dendritic cell; Mono, monocyte; PMN, polymorphonuclear neutrophils; NK cell, natural killer cell; NKT cell, natural killer T-cell. **b** The human *SIRPA* reference allele hSIRPαV1 is dominant in Europeans (EUR), Africans (AFR), Ad Mixed American (AMR) and South Asians (SAS), whereas hSIRPαV2 dominates in East Asians (EAS). Indicated percentages specify the *SIRPA* allele frequency of hSIRPαV1, hSIRPαV2 and hSIRPαV8. Not annotated, frequency > 3; Others, frequency < 3. **c** Sequence alignment of hSIRPαV1, hSIRPαV2, and hSIRPαV8 proteins (derived from [26]) demonstrates the differences within the CD47-binding extracellular Ig-like V-type (IgV) domain

It may be anticipated that targeting of the SIRPα/CD47 axis with an anti-SIRPα blocking mAb [25] displays a favorable safety profile due to the more restricted expression of SIRPα. SIRPα, SIRPβ and SIRPγ belong to the class of paired receptors comprising separate genes that encode proteins with similar extracellular regions but different transmembrane or cytoplasmic regions. These different regions have opposite (i.e. inhibitory or activating) signaling potentials. Both SIRPα and SIRPβ are expressed in myeloid lineage cells, while SIRPγ is expressed on T-cells, NK cells and NKT cells (Fig. 1a). SIRPγ binds to CD47 albeit with a 10-fold weaker affinity than SIRPα [27], whereas no ligand has been described for SIRPβ. The membrane distal extracellular Ig-like V-type (IgV) domain of SIRPα is highly polymorphic and thus far 10 human SIRPα alleles have been described [26]. In the present study, we report the development of ADU-1805, a potentially best-in-class pan-allele SIRPα mAb that blocks the interaction of SIRPα with CD47 and lacks binding to SIRPβ1. Targeting SIRPα enhanced tumor cell uptake by macrophages and neutrophils at a similar rate as anti-CD47 mAbs. Finally, we present that SIRPα blockade functionally differentiates from anti-CD47 mAbs and shows improved safety in vitro and in vivo.

## Methods

### Monoclonal antibody generation

Full-length cDNA of human SIRPα variant 1 (hSIRPαV1) (GenBank accession: NM_001040022.1) and hSIRPαV2 (GenBank accession: D86043.1) were synthesized (GeneArt, Thermo Fisher Scientific), subcloned into the pCI-neo vector (Promega) and used to immunize mice. Hybridomas were generated as described previously [28]. Selected stable hybridomas were cultured in serum-free media for 7 days, supernatants were harvested, and antibodies were purified using MabSelect Sure Protein A resin (GE Healthcare). Antibody concentrations were quantified using spectrophotometry. The isotype of antibodies was established using mouse a monoclonal antibody isotyping kit (Bio-Rad Laboratories).

### Antibody sequencing and expression

Antibody sequences were identified by DNA sequencing of the selected hybridomas (LakePharma). Antibody VH and VL genes were synthesized by GeneArt (Thermo Fisher Scientific), subcloned into the pcDNA3.1(+) vector (Thermo Fisher Scientific) and expressed in FreeStyle 293-F (Thermo Fisher Scientific) or ExpiCHO-S cells (Thermo Fisher Scientific). Transfected cells were cultured in serum-free media for 7 days and mAbs were purified using MabSelect Sure Protein A resin (GE Healthcare).

### Antibody humanization

Humanization of the mouse anti-human SIRPα.40A mAb (hSIRPα.40A) was performed by grafting complementarity-determining region (CDR) residues onto a human germline framework [29]. Differences between mouse hSIRPα.40A and the human framework residues were individually modeled by a homology model on basis of PDB ID 3UMT (light chain), PDB ID 1EHL (heavy chain) and PDB ID 3BGF (Fv) using Discovery Studio 4.5 (BIOVIA). Post-translational modification (PTM) motifs were removed where possible.

### Cell lines and cell culture

The BJAB (DSMZ), Raji (ECACC), THP-1 (ATCC), U-937 (ATCC) and NK-92MI (ATCC) human cell lines, the IC-21 (ATCC) mouse cell line, and the CHO-K1 (ATCC) hamster cell line were cultured as recommended by the vendor. Cell lines were validated as *Mycoplasma* negative by Baseclear B.V. (Leiden) using a validated PCR test.

### Antibody affinity measurement

A recombinant human SIRPα/His fusion protein (Sino Biological) was used for measuring monomeric binding affinity to hSIRPα.40A and derivatives thereof. Binding was assessed by bio-light interferometry (BLI) using amine coupling of mAbs to an AR2G biosensor (using standard NHS/EDC activation) followed by association/dissociation of recombinant hSIRPα/His and detection with the Octet RED96 (ForteBio).

### SIRPα binding and blocking assay

For binding ELISA, CHO-K1 cells were transiently transfected with pCI-neo vectors encoding human, mouse or cynomolgus monkey (*Macaca fascicularis*) SIRP genes. Transfected cells were incubated with indicated mAbs, bound antibodies were detected using goat-anti-mouse IgG-HRP conjugate (Southern Biotech) or goat-anti-rat IgG-HRP conjugate (Jackson ImmunoResearch), visualized with TMB Stabilized Chromogen (Invitrogen), and detected using an EnVision (PerkinElmer).

Binding of anti-human SIRPα mAbs to human SIRPγ was assessed by flow cytometry using NK-92MI cells. Antibodies were incubated at 4 °C, stained with AF647-labeled donkey anti-human IgG conjugate (Jackson ImmunoResearch), and analyzed by flow cytometry (FACSVerse, BD Biosciences).

SIRPα blocking ability was studied using THP-1 and U-937 AML cell lines, where after incubation with FcR Blocking Reagent (Miltenyi Biotec) and indicated mAbs, DyLight 488-labeled recombinant human CD47/Fc-protein (R&D Systems) was allowed to bind at 4 °C and analyzed by flow cytometry (FACSCanto II, BD Biosciences). SIRPα blocking ability on IC-21 cells was studied following incubation with indicated mAbs and recombinant mouse CD47/Fc-protein (R&D Systems) at 37 °C, detection of bound CD47 protein using anti-human IgG-HRP conjugate (Jackson ImmunoResearch), which was visualized with TMB Stabilized Chromogen (Invitrogen) and detected using an EnVision (PerkinElmer).

### Flow cytometry

Cells were phenotypically characterized using a FACSCanto II or FACSVerse flow cytometer with fluorochrome-conjugated mAbs (Additional file 1: Table S1). Further details can be found in Additional file 2: Extended methods.

### Primary cell isolation

Human blood was obtained from healthy volunteers who provided informed consent (Sanquin Bloodbank, Nijmegen, The Netherlands) and PBMCs were isolated by Ficoll-paque density gradient centrifugation. CD14+ monocytes were enriched (> 70% purity) using RosetteSep Human Monocyte Enrichment Cocktail (Stemcell Technologies). NK cells were enriched (> 90% purity) using the untouched human NK Cell Isolation Kit (Miltenyi Biotec).

Granulocytes were isolated from erythrocyte-depleted whole blood upon incubation with 10 ng/mL recombinant human interferon-γ (Immunotools) for 1 h at 37 °C in 5% CO_2_. Non-adherent blood cells were collected, and the percentage of granulocytes was determined by flow cytometry on the FACSCanto II (based on high FSC and SSC).

Similar procedures where applicable were also applied to EDTA whole blood obtained from healthy cynomolgus monkeys (Biomedical Primate Research Centre (BPRC), Rijswijk, The Netherlands).

### Neutrophil trogocytosis assay

Human Burkitt’s lymphoma BJAB cells were labeled with cell proliferation dye eFluor450 (Thermo Fisher Scientific). Labeled cells were mixed with assay medium (RPMI 1640 [Gibco], 10% fetal bovine serum [Gibco] and 100 IU/mL penicillin-streptomycin [Gibco]), indicated mAbs and 0.1 μg/mL rituximab (anti-hCD20), and were then added to human granulocytes (at a ratio of 1:1 tumor cell per phagocyte) and incubated at 37 °C in 5% CO_2_ for 2 h. Thereafter, 0.1 μg/mL propidium iodide was added to the mixture and trogocytosis (e.g. visualized as the appearance of eFluor450+ granulocytes) was assessed using the FACSVerse flow cytometer.

### Human macrophage generation and phagocytosis assay

Human macrophages were generated from CD14-enriched monocytes cultured in CellCarrier 96-well flat-bottom microplates (PerkinElmer) in medium (IMDM [Gibco], 8.5% fetal bovine serum [Gibco] and 100 IU/mL penicillin-streptomycin [Gibco]) containing 50 ng/mL human monocyte colony stimulating factor (M-CSF) for 7 days at 37 °C in 5% CO_2_. Raji cells labeled with eFluor450 were mixed with assay medium, indicated mAbs (anti-CD47 antibodies were used at 66.7 nM (10 μg/mL) and anti-SIRPα antibodies were titrated ranging from 66.7 nM (10 μg/mL) to 6.67 nM (1 μg/mL) and 0.67 nM (0.1 μg/mL)) and 1 μg/mL rituximab, and were then simultaneously added to human macrophages (at a ratio of 2.5:1 tumor cells per phagocyte) and incubated at 37 °C in 5% CO_2_ for 2 h. After washing and fixation with 2% formaldehyde, cells were stained with biotin-conjugated anti-human CD19 (eBioscience) for 1 h at room temperature (RT) and Alexa Fluor 488-conjugated streptavidin (Thermo Fisher Scientific). Nuclei were stained with DRAQ5 (Thermo Fisher Scientific) and phagocytosis was analyzed with the Operetta High-Content Imaging System (PerkinElmer). Data were processed and analyzed with Columbus V2.6 software (PerkinElmer). The phagocytosis of tumor cells was quantified counting at least 200 macrophages per sample and using an Uptake Index, as follows: (number of tumor cells inside macrophages/number of macrophages) × 100.

### Antibody-dependent cell-mediated cytotoxicity (ADCC) assay

CHO-K1.hSIRPαV1 cells were seeded in CellCarrier 384-well flat-bottom microplates (PerkinElmer) and indicated mAbs were added in assay medium together with human NK cells (at an effector:target cell ratio of 1:5). After overnight incubation at 37 °C in 5% CO_2_ cells were washed, stained with Fixable Viability Dye eFluor660 (Thermo Fisher Scientific) and fixed in 5% formalin for 10 min at RT. Fixed cells were washed and nuclei were stained with 1 μg/mL Hoechst 33342 (Life Technologies). Viable target cells were measured with the Operetta High-Content Imaging System and data were processed and analyzed with Columbus V2.6 software.

### Complement-dependent cytotoxicity (CDC) assay

Human U-937 leukemia cells were labeled with CellTrace CFSE dye (Thermo Fisher Scientific). Labeled U-937 cells were seeded in U-bottom 96-well plates, mixed with indicated mAbs and 20% human complement serum (Sigma-Aldrich) in assay medium, and incubated for 4 h at 37 °C in 5% CO_2_. Thereafter, 0.1 μg/mL DAPI was added to the mixture and CDC was assessed using the FACSVerse flow cytometer.

### Jurkat FcγRIIA-131H reporter assay

Antibody-mediated activation of FcγRIIA-131H was established using CHO-K1.hSIRPαV1 cells and Jurkat FcγRIIA-131H cells (Promega) at an effector:target cell ratio of 1:2, following the manufacturer’s instructions.

### Allogeneic mixed lymphocyte reaction (MLR)

To assess the allogeneic immune reaction, PBMCs from two human donors (referred to as the responder and stimulator (30 Gray (Gy) irradiated) were added together at a R:S ratio of 1.5:1) in presence of mAbs and incubated at 37 °C in 5% CO_2_ for 5 days. Supernatants were collected to quantify IFNγ levels by ELISA (Thermo Fisher Scientific). The remaining cells were stained with fluorescent mAbs against CD3, CD4, CD8, CD19 and CD56 for 30 min at 4 °C and analyzed by flow cytometry.

### SEB-induced T-cell proliferation

Human PBMCs were seeded in U-bottom 96-well plates, treated with 100 μg/mL of indicated mAbs and 1 μg/mL SEB (Sigma-Aldrich), and incubated for 3 days at 37 °C in 5% CO_2_. CD3 blast formation was assessed using the FACSVerse flow cytometer.

### Hemagglutination assay

EDTA-treated human whole blood collected from healthy donor volunteers was washed with PBS, a 1% erythrocyte suspension (v/v) was prepared in PBS, and 50 μL of the serially (2-fold) diluted mAbs or phytohemagglutinin (PHA-P; Sigma-Aldrich) were incubated with 50 μL of the 1% erythrocyte suspension for 2 h at RT in clear 96-well U-bottom plates. Hemagglutination (visible as loss of RBC “button” formation) was quantitated using the ChemiDoc Touch Imaging System and analyzed with Image Lab 5.2.1 software (Bio-Rad Laboratories).

### Platelet aggregation and activation assay

Blood was collected from healthy donor volunteers who provided informed consent (HaemoScan BV, Groningen, The Netherlands) and buffered with sodium citrate. To assess platelet aggregation (impedance method), blood was diluted with 0.9% NaCl continuously mixed with a stir bar, impedance electrodes were fixed into the blood-containing tubes, and indicated mAbs, adenosine 5′-diphosphate sodium salt (ADP; Sigma-Aldrich) or vehicle (10 mM L-Histidine pH 5.5 containing 0.1 M sodium chloride) were added to the blood suspension. Aggregation was measured for 6 min. The maximum slope of the aggregation curve for the first 3 min was determined from the recordings by “R: A language and environment for statistical computing” (R Foundation for Statistical Computing). To assess platelet activation, the blood suspension was incubated with indicated mAbs, arachidonic acid (Sigma-Aldrich) or vehicle for 1 h at 37 °C. Samples were centrifuged and plasma was collected to execute the thromboxane B2 enzyme immunoassay (Cayman Chemical).

### Cytokine release assay

Cytokine release was assessed on sodium heparin-preserved whole blood obtained from 24 healthy donor volunteers who provided informed consent (Sanquin Bloodbank, Nijmegen, The Netherlands). Indicated mAbs were added to polystyrene U-bottom 96-well plates, whole blood was added, and plates were incubated overnight at 37 °C in 5% CO_2_. The cytokines IL-6, IL-8, TNF-α, MCP-1, MIP-1α, and MIP-1β in the supernatants were detected using a custom human 6-plex assay kit (Thermo Fisher Scientific) and analyzed on the Bio-Plex MAGPIX multiplex reader (Bio-Rad Laboratories) equipped with Bio-Plex Manager 6.1 software (Bio-Rad Laboratories).

### Mouse tumor xenograft model

For tumor cell engraftment, 0.75 × 10^6^ Daudi cells (diluted 1:1 with Matrigel) were injected subcutaneously into the left flank of approximately 11-week-old NOD.Cg-*Prkdc*^*scid*^ *IL2rg*^tm1Wjl^/SzJ (NSG) mice, purchased from Charles River Laboratories (France). The animals were observed for tumor growth three times per week, starting 7 days after tumor cell injection. Treatment was initiated once tumors reached a size of 233 mm^3^ ± 78 mm^3^. Mice were given intravenous injections of 50 μg rituximab (anti-hCD20, human IgG1) or vehicle (0.9% NaCl) three times per week. In two of the groups that received rituximab, mice were given intraperitoneal injections of 500 μg anti-mSIRPα (clone .20A, mouse IgG1) three times per week, or alternatively mice were given daily intraperitoneal injections of 500 μg anti-hCD47 (clone B6H12, mouse IgG1) for a duration of 4 weeks. Mice were monitored for morbidity and mortality daily. Tumor size was measured three times per week and mice were sacrificed when tumor size reached 2000 mm^3^. Tumor sizes were measured using a digital caliper and tumor volumes in mm^3^ calculated with a modified ellipsoid formula: V = (length x width^2^) × 0.28. Animals were sacrificed when they reached humane endpoint or if they survived till day 34 after start of treatment.

### Toxicity study in NHPs

A single-dose toxicity study was conducted at Covance Preclinical Services GmbH (Münster, Germany) according to a written study protocol and facility standard operating procedures in compliance with Institutional Animal Care and Use Committee (IACUC) criteria, national legal regulations on animal welfare, and accepted animal welfare standards. All animals were experimentally naive, purpose-bred cynomolgus monkeys originating from Asia. For the single-dose study, male (*n* = 4) and female (*n* = 4) animals were administered a single 15-min intravenous (i.v.) infusion of ADU-1805 (0.3, 3 or 30 mg/kg) or vehicle control (10 mM L-Histidine pH 5.5 containing 0.1 M NaCl). In-life evaluations included clinical observations, body weight, food consumption, standard neurologic and cardiovascular safety pharmacology evaluations, clinical pathology (serum chemistry, hematology, and coagulation), and toxicokinetics. To assess the pharmacokinetic properties of ADU-1805 in cynomolgus monkey serum, blood was drawn on 0, 1, 8, and 24 h, and 3, 8, 11, 15, 22, 29, 36, 43, 59 days post single-dose ADU-1805. Fifty-nine days following the initial dose, the animals were necropsied and examined for gross observations, organ weights, and routine histopathologic evaluation was conducted on formalin-fixed paraffin embedded tissues collected at necropsy.

### Quantification and statistical analysis

Data are reported as mean ± standard deviation (SD) as specified. Statistical significance was determined by Student’s t-test or one-way analysis of variance (ANOVA) as indicated, using GraphPad Prism version 8 (CA, USA). All Student’s t-tests were two-sided under the assumption of equal variance between samples. All one-way ANOVA tests were corrected for multiple comparisons using statistical hypothesis testing. Differences were considered statistically significant if *p* < 0.05.

## Results

### Generation and characterization of a pan-SIRPα allele antibody

An unbiased single-nucleotide polymorphism (SNP) analysis of human SIRPα, based on data available at EnsEMBL (https://www.ensembl.org), revealed that SIRPαV1, SIRPαV2 and SIRPαV8 are the most prominent haplotypes present among the human population (Fig. 1b). Of these, SIRPαV1 and SIRPαV2 differ the most in their IgV domain sequence (Fig. 1c). While SIRPαV1 is the most abundant allele among European, Admixed American and African populations, the SIRPαV2 allele is the most commonly found allele in East Asian population.

**Fig. 2:**
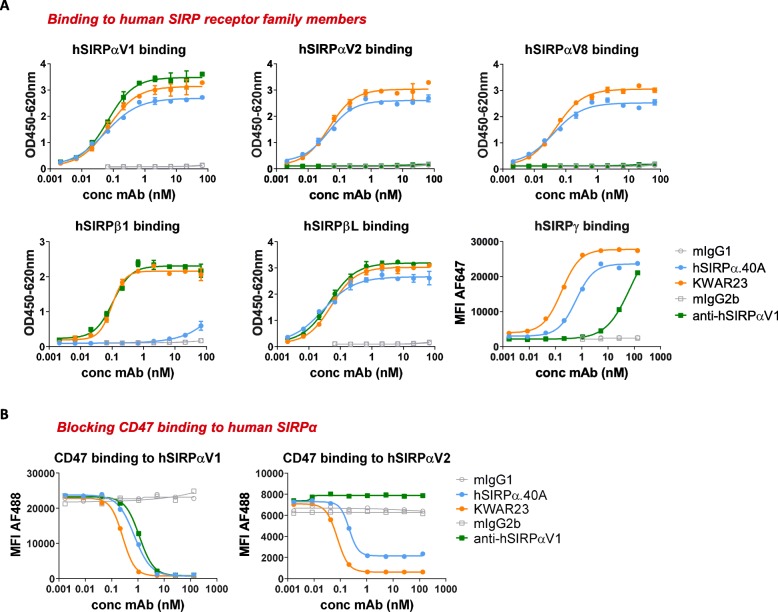
hSIRPα.40A is a CD47 blocking antibody with a unique epitope. **a** hSIRPα.40A shows pan-allele anti-hSIRPα binding, cross-reacts with hSIRPγ, and lacks appreciable hSIRPβ1 binding, thereby differentiating from allele-specific (anti-hSIRPαV1) and pan-hSIRP antibodies (KWAR23 [30]). **b** hSIRPα.40A blocks CD47 binding to hSIRPαV1 and hSIRPαV2-expressing U-937 and THP-1 AML cell lines. (**a**, **b**: Mean ± SD; representative of *n* = 2 is shown)

hSIRPα.40A was generated and identified as an antibody that demonstrated potent pan-allele SIRPα binding (i.e. binding human SIRPαV1, SIRPαV2 and SIRPαV8) and lacked appreciable SIRPβ1 binding (Fig. 2a). In contrast, the KWAR23 antibody binds to all SIRPα alleles and also the SIRPβ1 activating receptor. hSIRPα.40A and KWAR23 both bind human SIRPβL [31] and SIRPγ. hSIRPα.40A showed potent antagonism of the two most prevalent SIRPα alleles (e.g. SIRPαV1 and SIRPαV2), as determined by blockade of CD47 binding to U-937 and THP-1 AML cell lines that express SIRPαV1 (data not shown) and SIRPαV2 [32], respectively (Fig. 2b).

**Fig. 3:**
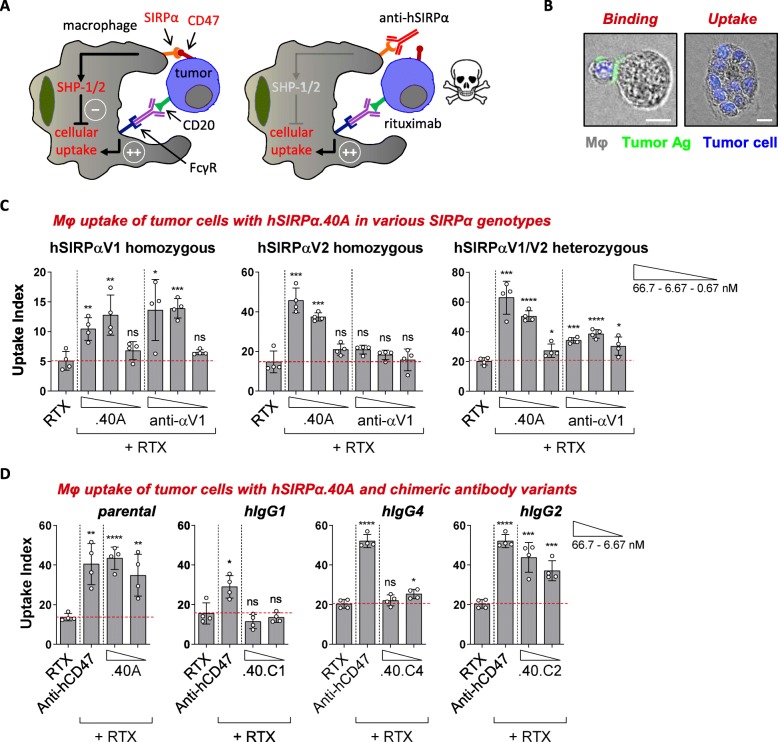
hSIRPα.40A promotes tumor cell uptake in all *SIRPA* genotypes. **a** Illustration of tumor cell uptake by human macrophages upon engagement of FcγR and blockade of the SIRPα/CD47 axis. **b** Picture showing a human macrophage binding to a Raji cell opsonized with anti-tumor antibodies (in green; left) resulting in tumor cell uptake (right). Scale bar, 10 μm. **c** hSIRPα.40A promotes rituximab (RTX)-mediated macrophage tumor cell uptake in both homozygous and heterozygous *SIRPA* genotypes. (Mean ± SD; representative of *n* = 2 (hSIRPαV1), 4 (hSIRPαV1/V2) or 6 (hSIRPαV2) donors is shown). **d** Chimeric hSIRPα.40A promotes optimal macrophage-mediated tumor cell uptake on a human IgG2 (.40.C2) but not on a IgG1 (.40.C1) or IgG4 (.40.C4) Fc backbone. (Mean ± SD; representative of *n* = 2 is shown). Data were analyzed by unpaired two-sided Student’s t-test. The asterisks (*) indicate statistical differences compared to the RTX control group: *p < 0.05, **p < 0.01, ***p < 0.001, ****p < 0.0001; ns, not significant

The functional activity of hSIRPα.40A was assessed in vitro using a macrophage-based phagocytosis assay (. 3a, b). In this assay human peripheral blood-derived macrophages that endogenously express SIRPα are co-incubated with Burkitt’s lymphoma Raji cells (expressing both CD20 and CD47 (Additional file 3: Figure S1A, B)). In presence of rituximab, hSIRPα.40A augmented tumor-cell uptake (calculated using the uptake index) of Raji cells by macrophages obtained from both *SIRPA* homozygous (SIRPαV1/SIRPαV1 and SIRPαV2/SIRPαV2) and heterozygous (SIRPαV1/SIRPαV2) individuals (Fig. 3c). The relevance of the unique binding profile of hSIRPα.40A was illustrated by the anti-hSIRPαV1 allele-specific mAb that only enhanced tumor cell phagocytosis by SIRPαV1/SIRPαV1 homozygous-derived macrophages while showing moderate or no phagocytosis by macrophages obtained from SIRPαV1/SIRPαV2 or SIRPαV2/SIRPαV2 individuals, respectively. Overall, this demonstrates the advantages of a pan-allele SIRPα antibody to all homozygous and heterozygous *SIRPA* individuals.

To evaluate the therapeutic effect of SIRPα blockade in vivo we generated the anti-mouse SIRPα surrogate mAb mSIRPα.20A that specifically bound mouse SIRPα, lacked cross-reactivity to SIRPβ, and blocked CD47 binding, similar to anti-mSIRPα clone p84 (Additional file 4: Figure S2A, B) [33]. Surrogate mAb mSIRPα.20A bound to all mouse *SIRPA* alleles, including NOD SIRPα which is able to bind to human CD47 (Additional file 5: Table S2) [26]. The ability of mSIRPα.20A to eliminate subcutaneously engrafted Daudi Burkitt’s lymphoma cells in NSG mice (that express the NOD *SIRPA* allele) was tested in combination with rituximab, analogous to the xenograft model described previously (Additional file 6: Figure S3A) [8]. Mice treated with the combination of mSIRPα.20A and rituximab showed decreased lymphoma burden and significantly prolonged survival compared to rituximab alone, confirming earlier observations (Additional file 6: Figure S3B, C) [25]. The blocking anti-hCD47 mAb B6H12 was taken along for comparison and showed complete inhibition of lymphoma engraftment when combined with rituximab. These results should be compared with caution as NSG mice lack an antigen sink for the anti-hCD47 antibody (e.g. anti-human CD47 does not bind to CD47 expressed on mouse cells).

### ADU-1805, humanized hSIRPα.40A

To allow for human use, the mouse parental hSIRPα.40A antibody was humanized**.** First, a chimeric version of hSIRPα.40A was generated by grafting the VH and VL sequences of hSIRPα.40A onto the human constant domains of an IgG1, IgG2 or IgG4 heavy chain and human kappa light chain, respectively (Fig. 3d). Although the parental hSIRPα.40A mAb augmented rituximab-induced phagocytosis of Raji cells by human macrophages similar to the anti-CD47 blocking mAb (AB6.12-IgG4PE), the activity of hSIRPα.40A was completely abrogated when its VH and VL sequences were grafted onto a human IgG1 or IgG4 Fc backbone. In contrast, the human IgG2 chimeric variant of hSIRPα.40A retained the activity of the mouse parental mAb. We hypothesized that the mAb Fc of the chimeric hSIRPα.40A interacted with FcγRs present on macrophages, which include at least the high affinity human IgG receptor FcγRI (CD64) and FcγRII (CD32) [34]. Indeed, human IgG1 and IgG4 variants of chimeric hSIRPα.40A bound to FcγRI while the human IgG2 variant did not (data not shown) [35]. In addition, human IgG1 and IgG4 Fc variants that minimize antibody Fc-FcγR interactions restored the enhancement of rituximab-mediated phagocytosis as compared to their wild-type counterparts (Additional file 7: Figure S4A), while similar mutations of the human IgG2 Fc did not further alter macrophage-dependent phagocytosis. Together, these data imply that an anti-SIRPα mAb should be grafted on a human IgG2 backbone to prevent engagement of FcγR on myeloid cells when bound to the antigen (creating a heterotrimeric interaction referred to as the ‘scorpion effect’ [36]) (Additional file 7: Figure S4B).

**Fig. 4:**
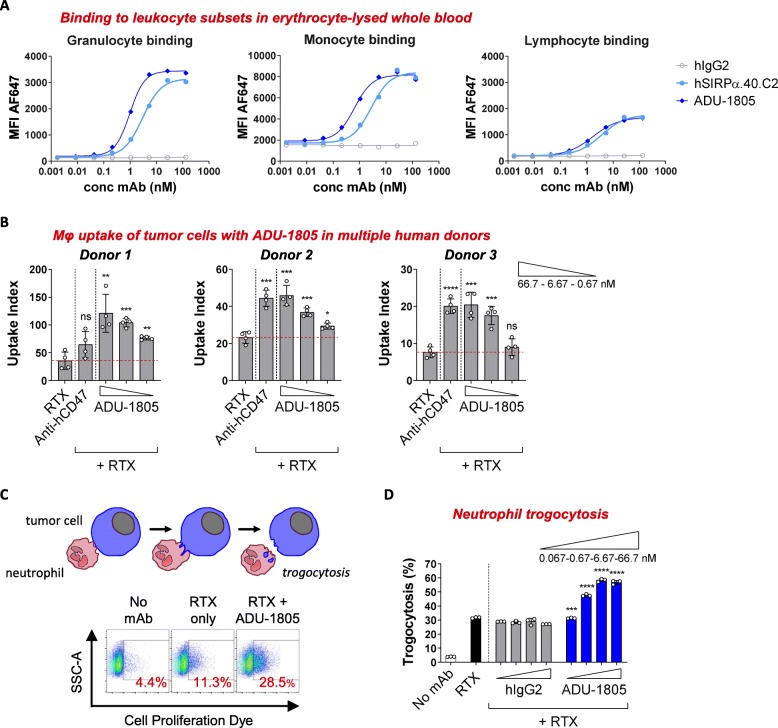
Antibody humanization and characterization of ADU-1805. **a** Binding of ADU-1805 to erythrocyte-depleted whole blood. (Mean; representative of *n* = 6 donors is shown). **b** ADU-1805 promotes macrophage-mediated tumor cell uptake, triggered by RTX. (Mean ± SD; representative of *n* = 7 donors is shown). Data were analyzed by unpaired two-sided Student’s t-test. * indicate statistical differences compared to the RTX control group: *p < 0.05, **p < 0.01, ***p < 0.001, ****p < 0.0001; ns, not significant. **c** The principle of tumor cell trogocytosis (*trogo* = nibble), a process by which neutrophils take small bites from target cells. Flow cytometry analysis demonstrates that ADU-1805 enhances anti-tumor antibody-induced trogocytosis in a dose-dependent manner. **d** Quantification of tumor cell trogocytosis by human neutrophils. (Mean ± SD; representative of *n* = 6 is shown). Data were analyzed by unpaired two-sided Student’s t-test. * indicate statistical differences compared to the respective isotype control group: ***p < 0.001, ****p < 0.0001

Subsequently, the mouse variable domains of hSIRPα.40A antibody were humanized by CDR grafting technology using matching human VH and VL frameworks [29], designated ADU-1805. ADU-1805 was confirmed to bind to monomeric human SIRPα antigen with a dissociation constant (KD) of 11 × 10^− 9^ M, similar to the parental and chimeric hSIRPα.40A mAb (Table 1). Moreover, ADU-1805 bound to SIRPα expressed on human monocytes (EC50 = 0.23–1.57 nM) and neutrophils (EC50 = 0.27–1.29 nM) but minimally bound to human lymphocytes (EC50 = 0.94–7.33 nM), which are known to express SIRPγ but not SIRPα [27] (Fig. 4a). Next, ADU-1805 was shown to enhance rituximab-induced phagocytosis, in a concentration-dependent manner, by human macrophages obtained from different human individuals (Fig. 4b). Also, ADU-1805 was shown to enhance rituximab-mediated cell killing by neutrophils in a concentration-dependent manner, through a process called trogocytosis [37] (Fig. 4c, d).

**Table 1:** hSIRPαV1 binding affinities of parental, chimeric, and humanized hSIRPα.40A variants measured on Octet AR2G biosensor. (Values represent Mean ± SD; *n* = 2–4 repeats)

Antibody	KD (M)	ka(1/Ms)	kdis(1/s)
hSIRPα.40A	12.1E-09 ± 3.7E-09	4.4E+ 05 ± 1.1E+ 05	5.6E-03 ± 3.0E-03
hSIRPα.40.C2	15.4E-09 ± 8.5E-11	4.4E+ 05 ± 9.7E+ 04	6.8E-03 ± 1.5E-03
ADU-1805	11.0E-09 ± 1.85E-09	3.4E+ 05 ± 8.7E+ 04	3.8E-03 ± 1.4E-03

### Differentiation between ADU-1805 and anti-CD47 agents

**Fig. 5:**
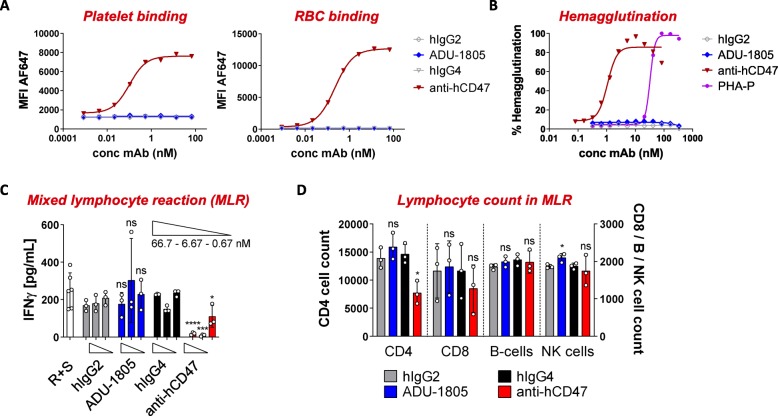
ADU-1805 is anticipated to have a favorable safety profile over CD47-targeting agents. **a** In contrast to anti-CD47 (AB6.12-IgG4PE), ADU-1805 does not bind to human platelets and RBCs, consistent with its binding specificities. (Mean; representative of *n* = 6 is shown). **b** ADU-1805 does not trigger hemagglutination. Anti-CD47 clone B6H12 and phytohemagglutinin (PHA-P) serve as positive control. (Mean; representative of *n* = 12 is shown). **c** ADU-1805 does not alter T-cell responses in an allogeneic MLR whereas anti-CD47 inhibits T-cell activation. The allogeneic immune reaction, when lymphocytes of two different donors are combined, results in T-cell activation. The resulting proliferation and/or production of cytokines were analyzed 5 days after start of culture. **d** Inhibition of T-cell activation by anti-CD47 coincides with a depletion of CD4+ T-cells. (**c**, **d**: Mean ± SD; representative of *n* = 3 donor combinations is shown). Data were analyzed by unpaired two-sided Student’s t-test. * indicate statistical differences compared to the respective isotype control group: *p < 0.05, ***p < 0.001, ****p < 0.0001; ns, not significant

The more restricted expression of SIRPα was hypothesized to allow SIRPα-targeting antibodies to differentiate from CD47-targeting agents. ADU-1805 lacked binding to human RBCs and platelets, and did not trigger hemagglutination, which is in line with its binding characteristics (Fig. 5a, b). Also, SIRPα-targeting with the chimeric hSIRPα.40A mAb did not induce platelet aggregation or activation (Additional file 8: Figure S5). The restricted expression of SIRPα was further demonstrated by comparing the reactivity of ADU-1805 and anti-CD47 towards human PBMCs. Anti-CD47 bound to all cell subsets present in the PBMC fraction (e.g. monocytes, B-cells, T-cells and NK cells), whereas ADU-1805 bound to monocytes and showed only minimal binding to T-cell subsets (Additional file 9: Figure S6). Altogether, based on the presented in vitro data, this confirms the hypothesis that ADU-1805 will show a biological activity profile differentiated from CD47-targeting agents by its more restrictive binding pattern (i.e. no antigen sink, minimal or no effect on RBCs and platelets).

A second potential differentiation was revealed by studying the effect of ADU-1805 on the role of CD47 in cell-cell adhesion through its interaction with SIRPγ on neighboring T-cells [27]. Piccio et al. have demonstrated that blocking the SIRPγ-CD47 interaction with specific antibodies against either CD47 or SIRPγ impaired T-cell activation by CD47+ antigen presenting cells [38]. Hence, we evaluated whether ADU-1805 affected T-cell activation in a PBMC-based allogeneic MLR. ADU-1805 did not alter the T-cell secretion of IFNγ triggered by the allogeneic MLR, whereas anti-CD47 mAb treatment inhibited IFNγ secretion (Fig. 5c). To understand the underlying cause of the reduced IFNγ secretion as seen for CD47-targeting mAbs, we characterized the immune cell subsets that were present at day 5. While the representation of the various cell types remained unchanged in the ADU-1805 and isotype control antibody conditions, anti-CD47 treatment reduced the number of CD4+ T-cells in comparison to its respective isotype control antibody (Fig. 5d). Similarly, we found that anti-CD47 also reduced activation and blast formation of CD4+ T-cells in a SEB-induced T-cell proliferation assay (Additional file 10: Figure S7A, B), whereas ADU-1805 did not appear to affect T-cell activation and proliferation.

### Preliminary assessment of ADU-1805 safety and pharmacokinetics

To complement the nonclinical antibody development, we demonstrated that ADU-1805 did not engage FcγRIIA, nor did it induce ADCC via FcγR-bearing NK cells (Additional file 11: Figure S8A, B). In addition, ADU-1805 did not induce CDC of the SIRPα-expressing U-937 AML cell line, consistent with the observation that human IgG2 is a poor C1q binder [39] (Additional file 11: Figure S8C). Furthermore, ADU-1805 did not induce cytokine secretion in human whole blood, similar to the FDA-approved human IgG2 antibody panitumumab targeting epidermal growth factor receptor (EGFR) (Additional file 12: Figure S9).

To assess the differentiation of ADU-1805, safety and pharmacokinetics (PK) of ADU-1805 were established in vivo, in a single dose intravenous infusion in cynomolgus monkeys (Table 2). First, two putative variants, SIRPαV1 (NM_001284750.1) and SIRPαV2 (XP_015313155.1) were identified in cynomolgus monkey, that share 99.2% sequence identity. These variants share a sequence identity of > 91% with human SIRPαV1 and SIRPαV2 and ADU-1805 bound to both cynomolgus variants with an EC50 ≤ 1 nM, similar to its binding affinity for human SIRPα (Additional file 13: Figure S10A). Furthermore, the ADU-1805 binding profile was comparable for human and cynomolgus monkey leukocytes (Additional file 13: Figure S10B).

**Table 2:** Study setup of the ADU-1805 non-GLP pilot toxicity study in 5–7 years old cynomolgus monkeys. A single dose of ADU-1805 or vehicle was administered i.v. for a duration of 15 min. Vehicle refers to the antibody formulation buffer: 10 mM L-Histidine pH 5.5 containing 0.1 M sodium chloride

Group	Treatment	Dose	Animals
1	Vehicle	–	1 male / 1 female
C2	ADU-1805	0.3 mg/kg	1 male / 1 female
3	ADU-1805	3 mg/kg	1 male / 1 female
4	ADU-1805	30 mg/kg	1 male / 1 female

**Fig. 6:**
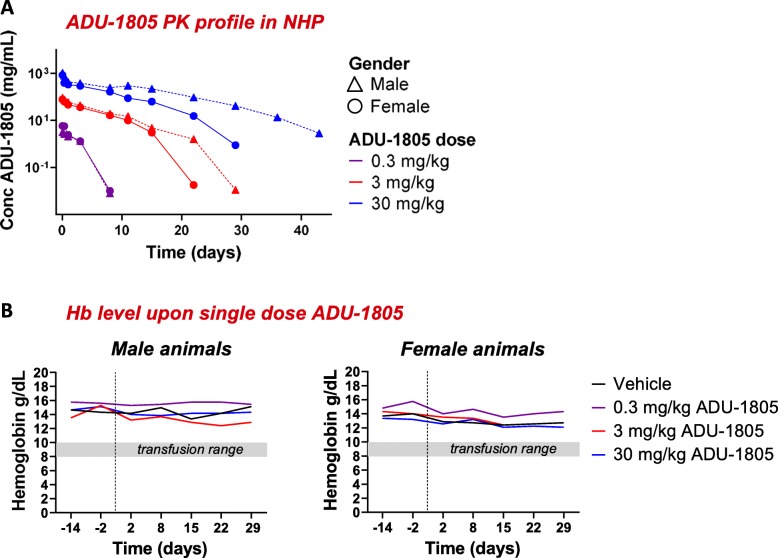
ADU-1805 can be safely administered intravenously in NHPs. **a** ADU-1805 single-dose pharmacokinetics profile in NHPs. Target-mediated drug disposition (TMDD) observed at the lowest dose. Dose proportional increase in exposure for the two higher dose levels (e.g. 3.0 mg/kg and 30 mg/kg). **b** ADU-1805 does not affect hemoglobin (Hb) levels in cynomolgus monkeys. Vertical dashed lines indicate infusion of monkeys on day 0. The shaded bar indicates the range of hemoglobin typically requiring a transfusion in humans [40]. (**a**, **b**: *n* = 6 animals)

Upon single-dose administration ADU-1805 measurements in serum followed by PK modelling demonstrated a dose proportional increase in exposure for the two higher dose levels with an estimated half-life of 1.86–6.41 days (Fig. 6a; Table 3). The administration of and exposure to ADU-1805 was well tolerated at all dose levels and no test-article related changes were observed. In contrast to the anti-CD47 mAb Hu5F9-G4 treatment-induced acute anemia in cynomolgus monkeys [40], none of the ADU-1805 doses affected the hemoglobin levels after single-dose administration. This finding supports that targeting SIRPα via ADU-1805 may have a favorable safety profile compared to CD47-targeting agents (Fig. 6b).

**Table 3:** The pharmacokinetic profile of ADU-1805 after a single dose exposure in NHPs

Dose	NCA half-life	Mid-range half-life
0.3 mg/kg	0.88 days	1.86 days
3 mg/kg	1.89 days	3.93 days
30 mg/kg	2.95 days	6.41 days

## Discussion

SIRPα-CD47 is considered an immune checkpoint (referred to as “don’t-eat-me”), similar to the well-established T-cell immune checkpoints (i.e. PD-1, CTLA-4), but is predominantly acting on cells of the myeloid lineage. A number of clinical trials are underway to evaluate SIRPα/CD47 blocking therapies [20–22], based on the notion that CD47 is overexpressed in various hematologic and solid tumors [6–11]. Blocking CD47 directly on tumor cells neutralizes the suppressive CD47 signal and activates macrophages through binding of the CD47-targeting agents to the FcγRs [41]. Also, macrophages recognize pro-phagocytic signals, such as calreticulin and phosphatidylserine that are induced on tumor cells as a result of therapies such as chemotherapy and radiotherapy [42], which in combination with inhibition of the SIRPα/CD47 axis are shown to promote tumor cell uptake. However, given the ubiquitous expression of CD47 on normal cells, on-target toxicity to healthy cells and a pronounced antigen sink present challenges with CD47-targeting approaches. Indeed, it has been observed that CD47-targeting agents (i.e. Hu5F9-G4, TTI-621) induce acute anemia and thrombocytopenia in patients [20, 22, 43] which may also further depend on the Fc format. Recently, two clinical trials evaluating anti-CD47 mAbs were terminated: CC-90002 in AML and myelodysplastic syndromes (MDS) and SRF231 in patients with advanced solid tumors and hematological cancers. In contrast, the acute toxicity initially observed with Hu5F9-G4 was ultimately managed by adopting a dosing strategy that involved a priming (1 mg/kg priming on day 1) and maintenance phase (30 mg/kg weekly for 3 doses and 30 mg/kg every other week thereafter) [40]. It remains to be seen whether this regimen will optimally induce anti-tumor activity. Next-generation variants of CD47 blocking agents such as ALX148 are being developed with reduced FcγR-binding properties [14]. Hence, ALX148 may induce reduced toxicity at the expense of single agent activity, similar to that seen with high-affinity SIRPα variants that lack an Fc chain [13]. Regardless, the broad expression of CD47 is thought to present an antigen sink on non-tumor tissue which remains a potential issue that could affect the bioavailability of the drug and thus its dosing strategy.

Due to its restricted tissue expression and predominant expression on cells of the myeloid lineage, direct targeting of SIRPα was hypothesized to overcome these CD47-targeting obstacles. Here, we describe a novel antagonistic pan-allele SIRPα antibody, hSIRPα.40A, and its humanized version ADU-1805. To assess the safety of SIRPα-targeting, we conducted a single-dose toxicity study in cynomolgus monkeys and did not observe obvious signs of toxicity with ADU-1805, in part illustrated by the stable hemoglobin levels in blood, and the lack of acute anemia and thrombocytopenia. The PK modelling of ADU-1805 in cynomolgus monkeys led to an estimated ADU-1805 half-life of 1.86–6.41 days in blood serum which is consistent with currently approved IgG2 backbone-based mAbs [44]. While the current toxicity study does not rule out a potential SIRPα antigen sink for ADU-1805, it is the first to provide evidence that selective SIRPα targeting may be a safe alternative for CD47-targeting agents.

Besides SIRPα, innate immune cells also express other inhibitory receptors such as sialic-acid-binding Ig-like lectin 10 (Siglec-10). Siglec-10 binds to CD24, a ligand that, like CD47, is overexpressed in multiple human cancers. Recent data demonstrated the therapeutic potential of CD24 blockade with monoclonal antibodies which promoted the phagocytic clearance of CD24+ cancer cells in vitro and in vivo [45]. A potential advantage of targeting CD24 instead of CD47 is its absence on RBCs. However, CD24 is also present on B-cells, neutrophils, neurons and epithelial cells, and healthy B-cells are efficiently phagocytosed by macrophages upon anti-CD24 therapy. The expression of Siglec-10 is also not restricted to macrophages [46]. The above raises the concern of antigen sink and/or safety issues due to targeting of the Siglec-10–CD24 axis.

ADU-1805 is designed to bind to all described human SIRPα alleles and block CD47 binding without cross-reacting to SIRPβ1, thereby differentiating it from other anti-SIRPα mAbs currently in preclinical development (i.e. BI 765063, KWAR23). In addition, ADU-1805 also binds to SIRPγ expressed on T-cells, albeit with a 2.9-fold reduced EC50 compared to SIRPα, and thus may block the SIRPγ-CD47 interaction. Targeting of this interaction using anti-CD47 mAbs or an anti-SIRPγ mAb, was previously shown to inhibit T-cell proliferation in an allogeneic MLR [38, 47, 48]. We therefore assessed whether ADU-1805 alters T-cell activation using a similar assay. Despite minimal binding to T-cells, ADU-1805 did not affect T-cell activation in an allogeneic MLR. ADU-1805 also did not change SEB-induced T-cell proliferation. In contrast, anti-CD47 mAb treatment had an inhibitory effect in both T-cell activation assays. We observed that anti-CD47 treatment ex vivo mainly decreased the number of (activated) CD4+ T-cells, while the effect on CD8+ T-cells was minimal. These results are in line with the defect of CD47^−/−^ CD4+ T-cells, that do respond to T-cell receptor (TCR)-induced activation, but exhibit a premature block in proliferation and survival [49]. It is unclear whether the reduced T-cell responsiveness recorded for CD47-targeting agents translates to clinic administration. So, while encouraging early responses (i.e. tumor shrinkage by means of macrophage phagocytosis) with anti-CD47 have been observed in patients [20], longer follow-up is needed to address T-cell activation, since this may be required for durability of clinical responses.

We showed that FcγR binding of hSIRPα.40A inhibits its functionality, which effect was absent by selection of a human IgG2 Fc-tail. Here, it is hypothesized that once anti-SIRPα binds to its target on FcγR-bearing myeloid cells it may simultaneously co-engage activating or inhibitory FcγRs on the same cell, thereby creating a heterotrimeric interaction (Additional file 7: Figure S4B). This so-called scorpion effect [36] could modulate the therapeutic effect of an antibody. Similar observations were made for antibodies directed against colony-stimulating factor 1 receptor (CSF1R), where for H27K15, a non-ligand competitive anti-CSF1R mAb [50], it was shown that the Fc region participates in its mode of action, suggestive of a similar scorpion effect.

Our data suggest that SIRPα targeting by ADU-1805 can activate myeloid cell types such as neutrophils and macrophages. The role of neutrophils and macrophages upon SIRPα blockade is further confirmed in xenograft mouse models that are deficient in T-cells, B-cells and NK cells [25, 30]. Additional preclinical studies in syngeneic mouse models demonstrate that anti-SIRPα monotherapy changes the composition of immune cells in the tumor microenvironment with an apparent increase in the number of M1 type macrophages and a concomitant decrease in the M2 type [25]. TAMs are thought to differentiate predominantly into those of the M2 type, which display pro-tumorigenic activity and are implicated in the abrogation of anti-tumor immunity [51]. Repolarization of TAMs into M1 type macrophages may skew the microenvironment towards becoming more pro-inflammatory thereby promoting the anti-tumor immune response. Moreover, anti-SIRPα treatment led to a marked increase in the number of tumor-infiltrating NK cells and CD8+ T-cells, and antibody-mediated depletion of these cells decreased the inhibitory effect of SIRPα blockade on tumor formation [25]. Finally, anti-SIRPα mAbs have the ability to enhance the activity of immune checkpoint inhibitors such as anti-PD-1 [25] and this has been confirmed in vivo with the CD47-blocking molecule ALX148 in combination with anti-PD-1 or anti-PD-L1 therapy [14]. The enhanced anti-tumor response with agents blocking the SIRPα-CD47 interaction may arise from the activation of multiple DC subsets (i.e. shown by increased CD86 expression) that is seen within the spleen (data not shown) [14]. Consequently, blockade of the SIRPα/CD47 axis increases adaptive immune responses in combination with immune checkpoint inhibitors. Taken together, this suggests that agents targeting the SIRPα–CD47 innate immune checkpoint induce anti-tumor immunity by bridging innate and adaptive immune responses. Thus, we believe that blockade of the SIRPα/CD47 axis using a pan-allele SIRPα mAb provides a novel approach to immunotherapy that may be applicable for a broad range of cancers. Nevertheless, it will be crucial to perform SIRPα SNP analysis and also biomarker analysis of treated patients in clinical trials. In the end, such retrospective studies could help to differentiate a predictive signature based on responders and non-responders.

## Conclusions

ADU-1805 is a potentially best-in-class antagonistic SIRPα-targeting antibody with a unique epitope that encompasses pan-allele SIRPα binding. Unlike anti-CD47 mAbs, ADU-1805 does not trigger depletion of RBCs and platelets when tested at increasing dose levels in NHPs, supporting its favorable safety profile. The data presented herein support further development of ADU-1805.

## Supplementary information

**Additional file 1:** **Table S1.** Antibodies used in this study.

**Additional file 2.** Extended methods.

**Additional file 3:** **Figure S1.** CD20 and CD47 expression in human Burkitt’s lymphoma cell lines.

**Additional file 4: Figure S2.** Specificity of anti-mouse SIRPα antibodies.

**Additional file 5: Table S2.** Characteristics of the anti-mSIRPα antibodies described in this study. Mouse-rat chimeric mSIRPα.20A was generated by grafting the cDNA encoding the heavy chain and light chain variable domains onto the constant mouse IgG1 heavy chain and mouse kappa light chain.

**Additional file 6** **Figure S3.** Anti-mSIRPα improves the efficacy of rituximab in NSG mice.

**Additional file 7:** **Figure S4.** Tumor cell uptake with anti-hSIRPα is most effective in absence of FcγR binding.

**Additional file 8:** **Figure S5.** Chimeric hSIRPα.40A does not impair platelet function.

**Additional file 9:** **Figure S6.** Anti-hSIRPα has a more selective binding profile as compared to anti-CD47.

**Additional file 10:** **Figure S7.** Anti-hSIRPα does not impair CD4+ or CD8+ T-cell proliferation.

**Additional file 11:** **Figure S8.** ADU-1805 is devoid of immune effector functions exemplified in complement and FcγR-dependent assays.

**Additional file 12:** **Figure S9.** ADU-1805 does not induce cytokine release in human whole blood.

**Additional file 13:** **Figure S10.** Cross-reactivity of ADU-1805 to cynomolgus monkey SIRPα.

## Data Availability

All data generated that are relevant to the results presented in this article are included in this article and its supplementary files (Additional files). Other data that were not relevant for the results presented here are available from the corresponding author upon reasonable request.

## Abbreviations

ADCC: Antibody-dependent cell-mediated cytotoxicity
ADCP: Antibody-dependent cellular phagocytosis
BLI: Bio-light interferometry
CD: Cluster of differentiation
CDC: Complement-dependent cytotoxicity
CDR: Complementarity-determining region
CELISA: Cell-based ELISA; CHO: Chinese hamster ovary
CSF1R: Colony-stimulating factor 1 receptor
Fc: Fragment crystallizable
FcγR: Fcγ receptor
IgG: Immunoglobulin G
IL: Interleukin
mAb: monoclonal antibody
M-CSF: Macrophage colony-stimulating factor
MFI: Mean fluorescence intensity
MHC: Major histocompatibility complex
MLR: Mixed lymphocyte reaction
NHP: Non-human primate
PBMC: Peripheral blood mononuclear cells
PD-1: Programmed cell death-1
PD-L1: Programmed death-ligand 1
PK: Pharmacokinetics
PTM: Post-translational modification
RBC: Red blood cell
SEB: *Staphylococcus* enterotoxin B
SIRPα: Signal-regulatory protein α
TAM: Tumor-associated macrophage
VH: Variable domain heavy chain
VL: Variable domain light chain
