# Supplementary material for: Functional characterization of the selective pan-allele anti-SIRPα antibody ADU-1805 that blocks the SIRPα–CD47 innate immune checkpoint
Source: J Immunother Cancer. 2019 Dec 4;7:340. doi: 10.1186/s40425-019-0772-0 (PMC6894304; doi:10.1186/s40425-019-0772-0)
Supplement: Supplementary file 1 — Additional file 1: Table S1. Antibodies used in this study. [file 40425_2019_772_MOESM1_ESM.docx]

**Additional file 1: Table S1.** Antibodies used in this study.

| ***Unconjugated antibodies reactive to human*** | | | | | | |
| --- | --- | --- | --- | --- | --- | --- |
| **Antigen** | **Reactivity** | **Host** | **Clone** | **Manufacturer** | **Cat.#** | **Conjugate** |
| Alemtuzumab (Lemtrada) | human | human | Campath-1H | Genzyme | - | - |
| CD16 | human | mouse | 3G8 | eBioscience | 16-0166-82 | - |
| CD20 | human | mouse | 2B8 | InvivoGen | hcd20-mab1 | - |
| CD20 | human | mouse | 2B8 | InvivoGen | hcd20-mab9 | - |
| CD32 | human | mouse | 6C4 | eBioscience | 16-0329-81 | - |
| CD47 | human | human | AB6.12-IgG4PE | Evitria AG | - | - |
| CD47 | human | human | B6H12 | Evitria AG | - | - |
| CD64 | human | mouse | 10.1 | eBioscience | 16-0649-81 | - |
| CD172a (SIRPαV1) | human | mouse | 602411 | R&D Systems | MAB4546 | - |
| CD172a (pan-SIRPα) | human | mouse | KWAR23 | Evitria AG | - | - |
| FcR blocking reagent | human | - | - | Miltenyi Biotec | 130-059-901 | - |
| hIgG1 isotype control | - | human | - | Bio-Rad | PHP010 | - |
| hIgG1 isotype control | - | human | - | BioLegend | 403102 | - |
| hIgG2 isotype control | - | human | - | Sigma-Aldrich | I5404 | - |
| hIgG4 isotype control | - | human | - | Sigma-Aldrich | I4639 | - |
| HLA class I | human | mouse | W6/32 | InvivoGen | hla-c1 | - |
| mIgG1 isotype control | - | mouse | P3.6.2.8.1 | eBioscience | 16-4714-85 | - |
| mIgG2b isotype control | - | mouse | eBMG2b | eBioscience | 16-4732-85 | - |
| rIgG1 isotype control | - | rat | eBRG1 | eBioscience | 16-4301 | - |
| rIgG2a isotype control | - | rat | eBR2a | eBioscience | 14-4321-85 | - |
| Panitumumab (Vectibix) | human | human | E7.6.3 | Amgen | 1014135 | - |
| Rituximab (MabThera) | human | mouse | 2B8 | Mediq pharmacy | - | - |
| ***Unconjugated antibodies reactive to mouse*** | | | | | | |
| CD172a (pan-SIRPα) | mouse | rat | p84 | BioLegend | 144002 | - |
| CD47 | human | mouse | B6H12 | Bio X Cell | BE0019-1 | - |
| ***Conjugated antibodies reactive to human*** | | | | | | |
| **Antigen** | **Reactivity** | **Host** | **Clone** | **Manufacturer** | **Cat.#** | **Conjugate** |
| CD3 | human | mouse | UCHT-1 | BD Biosciences | 555333 | PE |
| CD3 | human | mouse | UCHT-1 | BD Biosciences | 561807 | FITC |
| CD4 | human | mouse | RPA-T4 | BD Biosciences | 555349 | APC |
| CD4 | human | mouse | RPA-T4 | BD Biosciences | 560158 | APC-H7 |
| CD8 | human | mouse | HIT8a | BD Biosciences | 555634 | FITC |
| CD8 | human | mouse | RPA-T8 | BD Biosciences | 560774 | V500 |
| CD14 | human | mouse | M5E2 | BD Biosciences | 557742 | PE-Cy7 |
| CD14 | human | mouse | 63D3 | BioLegend | 367108 | APC-Cy7 |
| CD16 | human | mouse | 3G8 | BD Biosciences | 556619 | PE |
| CD16 | human | mouse | NKP15 | BD Biosciences | 335035 | FITC |
| CD19 | human | mouse | SJ25C1 | BD Biosciences | 557791 | ACP-Cy7 |
| CD19 | human | mouse | HIB19 | BioLegend | 302230 | PerCP-Cy5.5 |
| CD19 | human | mouse | HIB19 | eBioscience | 13-0199-82 | Biotin |
| CD25 | human | mouse | M-A251 | BD Biosciences | 555434 | APC |
| CD32 | human | mouse | 3D3 | BD Biosciences | 552883 | FITC |
| CD41 | human | mouse | HIP8 | BioLegend | 303716 | APC-Cy7 |
| CD56 | human | mouse | MEM-188 | BioLegend | 304628 | PE-Cy7 |
| CD56 | human | mouse | B159 | BD Biosciences | 557747 | PE-Cy7 |
| CD64 | human | mouse | 22 | Beckman Coulter | IM1604U | FITC |
| CD172a/b (SIRPα/β) | human | mouse | SE5A5 | BioLegend | 323806 | PE |
| CD235a | human | mouse | GA-R2 (HIR2) | BD Biosciences | 562938 | BV421 |
| mIgG1 isotype control | - | mouse | MOPC-21 | BD Biosciences | 559320 | PE |
| mIgG1 isotype control | - | mouse | X40 | BD Biosciences | 345815 | FITC |
| ***Conjugated antibodies cross-reactive to cynomolgus*** | | | | | | |
| **Antigen** | **Reactivity** | **Host** | **Clone** | **Manufacturer** | **Cat.#** | **Conjugate** |
| CD3 | human, cyno | mouse | SP34-2 | BD Biosciences | 562877 | BV421 |
| CD4 | human, cyno | mouse | L200 | BD Biosciences | 550631 | PerCP |
| CD8 | human, cyno | mouse | RPA-T8 | BD Biosciences | 557086 | PE |
| CD14 | human, cyno | mouse | M5E2 | BD Biosciences | 557742 | PE-Cy7 |
| CD20 | human, cyno | mouse | 2H7 | BD Biosciences | 560853 | APC-H7 |
| ***Secondary antibodies*** | | | | | | |
| **Antigen** | **Reactivity** | **Host** | **Clone** | **Manufacturer** | **Cat.#** | **Conjugate** |
| Donkey anti-human IgG | human | donkey | - | Jackson Immuno Research | 709-605-149 | AF647 |
| Goat anti-human IgG | human | goat |  | Southern Biotech | 2040-02 | FITC |
| Goat anti-human IgG F(ab) | human | goat | - | Jackson Immuno Research | 109-007-003 | - |
| Goat anti-mouse Ig | mouse | goat | - | BD Biosciences | 349031 | FITC |
| Goat anti-mouse IgG F(ab')2 | mouse | goat | - | Thermo Fisher Scientific | A11017 | AF488 |
| Goat anti-mouse IgG F(ab')2 | mouse | goat | - | Thermo Fisher Scientific | A-21237 | AF647 |
| Streptavidin | - | - | - | Thermo Fisher Scientific | S32354 | AF488 |
| Donkey anti-human IgG-HRP | human | donkey | - | Jackson Immuno Research | 709-035-149 | HRP |
| Goat anti-mouse IgG-HRP | mouse | goat | - | Southern Biotech | 1030-05 | HRP |
| Goat anti-rat IgG-HRP | rat | goat | - | Jackson ImmunoResearch | 112-035-167 | HRP |
