# Supplementary material for: Functional characterization of the selective pan-allele anti-SIRPα antibody ADU-1805 that blocks the SIRPα–CD47 innate immune checkpoint
Source: J Immunother Cancer. 2019 Dec 4;7:340. doi: 10.1186/s40425-019-0772-0 (PMC6894304; doi:10.1186/s40425-019-0772-0)
Supplement: Supplementary file 2 — Additional file 2. Extended methods. [file 40425_2019_772_MOESM2_ESM.docx]

**Additional file 2: Extended methods.**

**Flow cytometry**

Human granulocytes, monocytes and lymphocytes present in erythrocyte-depleted whole blood were distinguished based on forward scatter (FSC) and side scatter (SSC). Erythrocytes (whole blood) and platelets (erythrocyte-depleted whole blood) were separated based on FSC/SSC and stained with mAbs against CD235a and CD41, respectively. Human immune cell subsets present in PBMCs were separated based on CD3 (T-cells), CD4, CD8, CD14 (monocytes), CD19 (B-cells), and CD56 (NK cells). Cynomolgus immune cell subsets present in erythrocyte-depleted whole blood were separated based on CD3 (T-cells), CD4, CD8, CD14 (monocytes), and CD20 (B-cells) using anti-human antibodies that are cross-reactive to cynomolgus monkey. Granulocytes were distinguished based on FSC and SSC, and CD3- CD20- lymphocytes were considered as NK cells. Cell viability was assessed by DAPI, propidium iodide (BD Biosciences) or Fixable Viability Dye eFluor660 (Thermo Fisher Scientific) staining as indicated. All flow cytometry data were analyzed using FlowJo v10 software (FlowJo, LLC).
