# Supplementary material for: Functional characterization of the selective pan-allele anti-SIRPα antibody ADU-1805 that blocks the SIRPα–CD47 innate immune checkpoint
Source: J Immunother Cancer. 2019 Dec 4;7:340. doi: 10.1186/s40425-019-0772-0 (PMC6894304; doi:10.1186/s40425-019-0772-0)
Supplement: Supplementary file 3 — Additional file 3: Figure S1. CD20 and CD47 expression in human Burkitt’s lymphoma cell lines. [file 40425_2019_772_MOESM3_ESM.pdf]

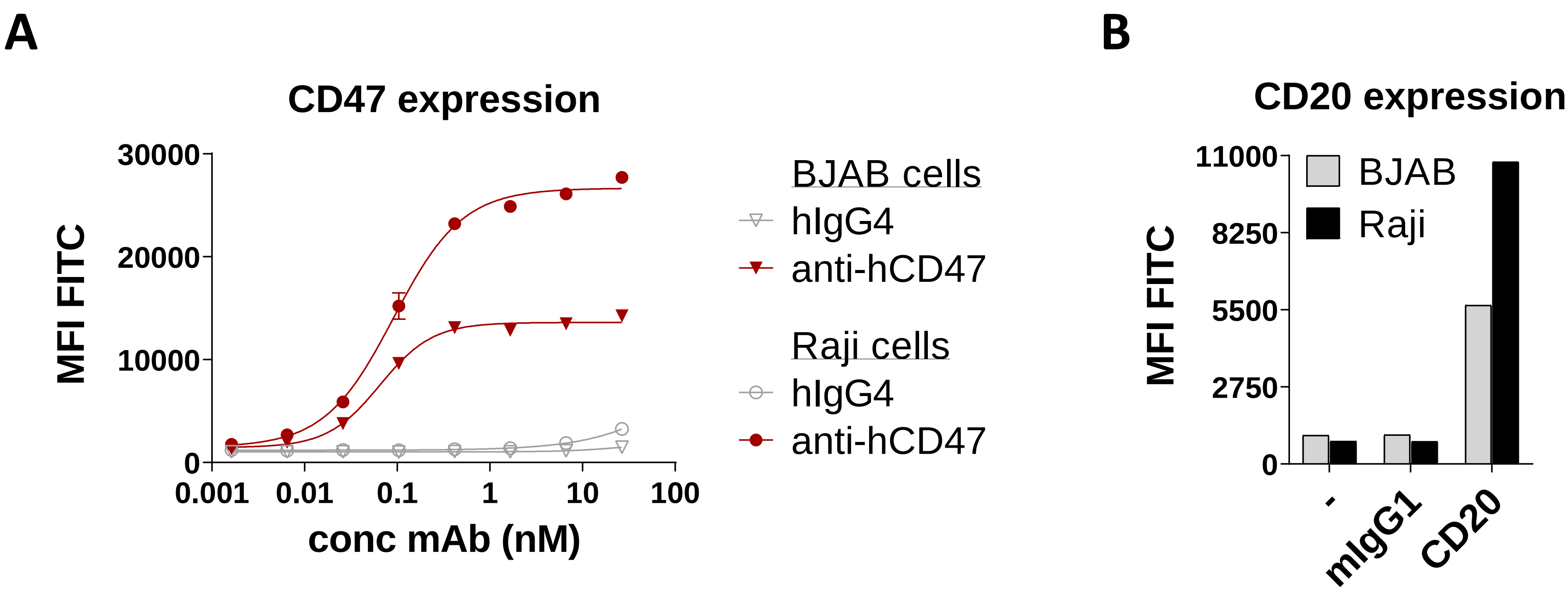

Additional file 3: Figure S1. CD20 and CD47 expression in human Burkitt's lymphoma cell lines.
(**A**) CD47 expression was determined in BJAB and Raji human human Burkitt’s lymphoma cell lines. (Mean ± SD is shown). (**B**) CD20 expression was determined in BJAB and Raji human Burkitt’s lymphoma cell lines.
