# Supplementary material for: Functional characterization of the selective pan-allele anti-SIRPα antibody ADU-1805 that blocks the SIRPα–CD47 innate immune checkpoint
Source: J Immunother Cancer. 2019 Dec 4;7:340. doi: 10.1186/s40425-019-0772-0 (PMC6894304; doi:10.1186/s40425-019-0772-0)
Supplement: Supplementary file 4 — Additional file 4: Figure S2. Specificity of anti-mouse SIRPα antibodies. [file 40425_2019_772_MOESM4_ESM.pdf]

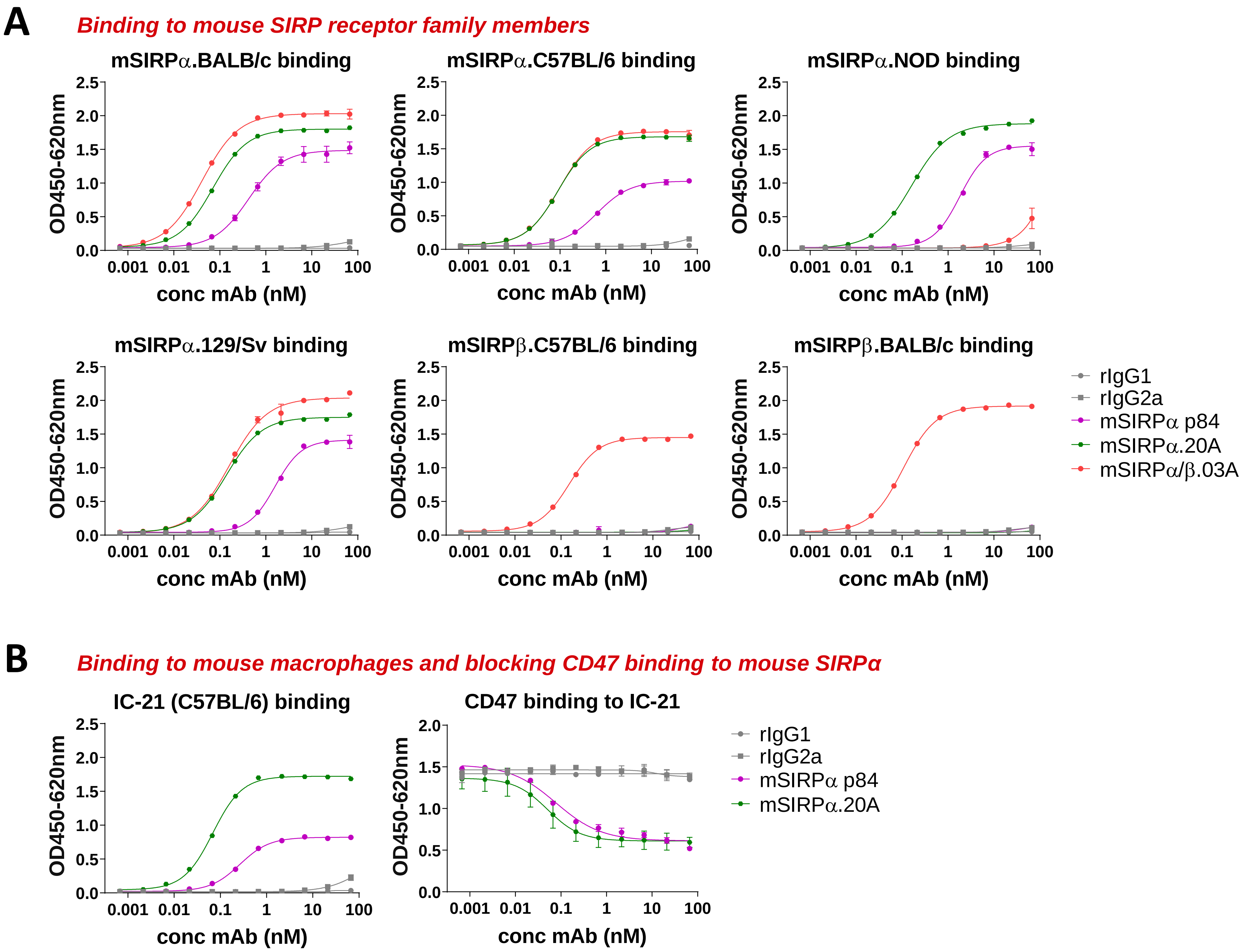

Additional file 4: Figure S2. Specificity of anti-mouse SIRPα antibodies.
(A) Hybridoma-purified anti-mSIRPα antibodies selectively bind mouse SIRPα transiently expressed on CHO-K1 cells without obvious cross-reactivity towards mouse SIRPβ. (B) Anti-mSIRPα antibodies bind to the C57BL/6 mouse-derived peritoneal macrophage cell line IC-21 and block CD47 binding. (A, B: Mean ± SD; representative of n = 2 is shown).
