# Supplementary material for: Functional characterization of the selective pan-allele anti-SIRPα antibody ADU-1805 that blocks the SIRPα–CD47 innate immune checkpoint
Source: J Immunother Cancer. 2019 Dec 4;7:340. doi: 10.1186/s40425-019-0772-0 (PMC6894304; doi:10.1186/s40425-019-0772-0)
Supplement: Supplementary file 5 — Additional file 5: Table S2. Characteristics of the anti-mSIRPα antibodies described in this study. Mouse-rat chimeric mSIRPα.20A was generated by grafting the cDNA encoding the heavy chain and light chain variable domains onto the constant mouse IgG1 heavy chain and mouse kappa light chain. [file 40425_2019_772_MOESM5_ESM.docx]

**Additional file 5: Table S2.** Characteristics of the anti-mSIRPα antibodies described in this study. Mouse-rat chimeric mSIRPα.20A was generated by grafting the cDNA encoding the heavy chain and light chain variable domains onto the constant mouse IgG1 heavy chain and mouse kappa light chain.

| **Antibody** | **Strain reactivity** | **Fc format** | **Binding domain** | **Blocking activity** |
| --- | --- | --- | --- | --- |
| mSIRPα p84 | BALB/c  C57BL/6  NOD  129/Sv | ratIgG1 | IgC1 | Partial |
| mSIRPα.20A | BALB/c  C57BL/6  NOD  129/Sv | ratIgG2a; mIgG1 chimeric | IgV | partial |
| mSIRPα/β.03A | BALB/c  C57BL/6  129/Sv | ratIgG2a | IgV | full |
