# Supplementary material for: Functional characterization of the selective pan-allele anti-SIRPα antibody ADU-1805 that blocks the SIRPα–CD47 innate immune checkpoint
Source: J Immunother Cancer. 2019 Dec 4;7:340. doi: 10.1186/s40425-019-0772-0 (PMC6894304; doi:10.1186/s40425-019-0772-0)
Supplement: Supplementary file 6 — Additional file 6 Figure S3. Anti-mSIRPα improves the efficacy of rituximab in NSG mice. [file 40425_2019_772_MOESM6_ESM.pdf]

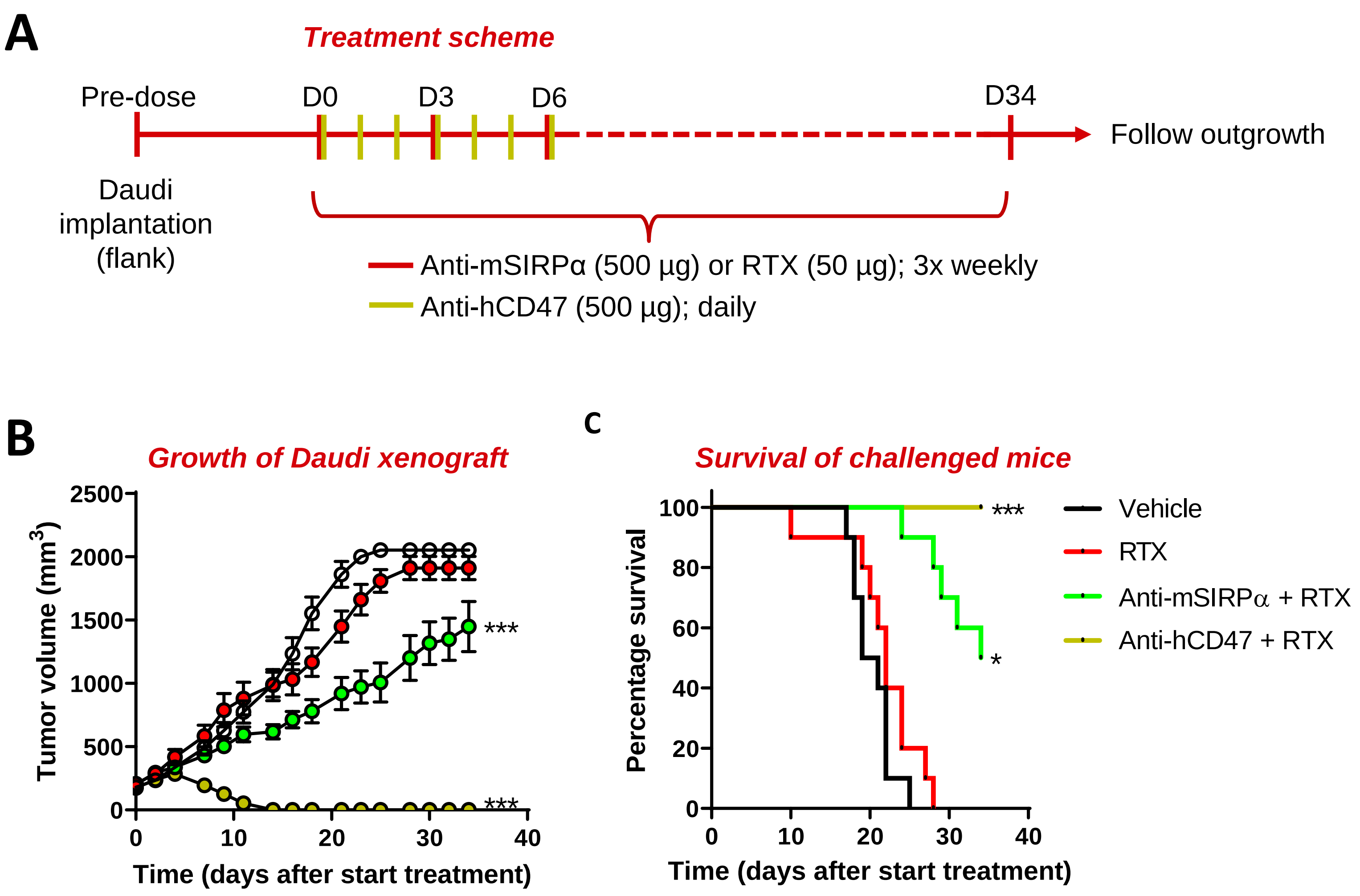

Additional file 6: Figure S3. Anti-mSIRPα improves the efficacy of rituximab in NSG mice. (**A**) Treatment schedule depicting the moment of tumor cell engraftment (day 0), antibody treatment, and tumor outgrowth until the moment of sacrifice (tumor volume ≥ 2000 mm^3). (**B**) Anti-mSIRPα inhibits the growth of Daudi tumor cells. NSG mice were subcutaneously engrafted with $0.75*10^6$ Daudi (human Burkitt's lymphoma) cells. Once tumors reached a size of 233 mm^3 ± 78 mm^3 mice were administered three times per week with 50 µg rituximab (RTX) alone or in combination with 500 µg anti-mSIRPα by intraperitoneal injection. Alternatively, RTX was combined with a daily administration of 500 µg anti-hCD47 (B6H12) which served as a positive control. (Mean ± SEM; *n* = 10 animals per group). (**C**) Anti-mSIRPα improves overall survival of Daudi-engrafted NSG mice when combined with a suboptimal dose of RTX. The vehicle refers to sterile saline (0.9% NaCl). Data were analyzed by Fisher's exact test with a one-sided critical region and a Bonferroni correction. * indicate statistical differences compared to the vehicle control group: *$p < 0.05$, ***$p < 0.001$; ns, not significant.
