# Supplementary material for: Functional characterization of the selective pan-allele anti-SIRPα antibody ADU-1805 that blocks the SIRPα–CD47 innate immune checkpoint
Source: J Immunother Cancer. 2019 Dec 4;7:340. doi: 10.1186/s40425-019-0772-0 (PMC6894304; doi:10.1186/s40425-019-0772-0)
Supplement: Supplementary file 7 — Additional file 7: Figure S4. Tumor cell uptake with anti-hSIRPα is most effective in absence of FcγR binding. [file 40425_2019_772_MOESM7_ESM.pdf]

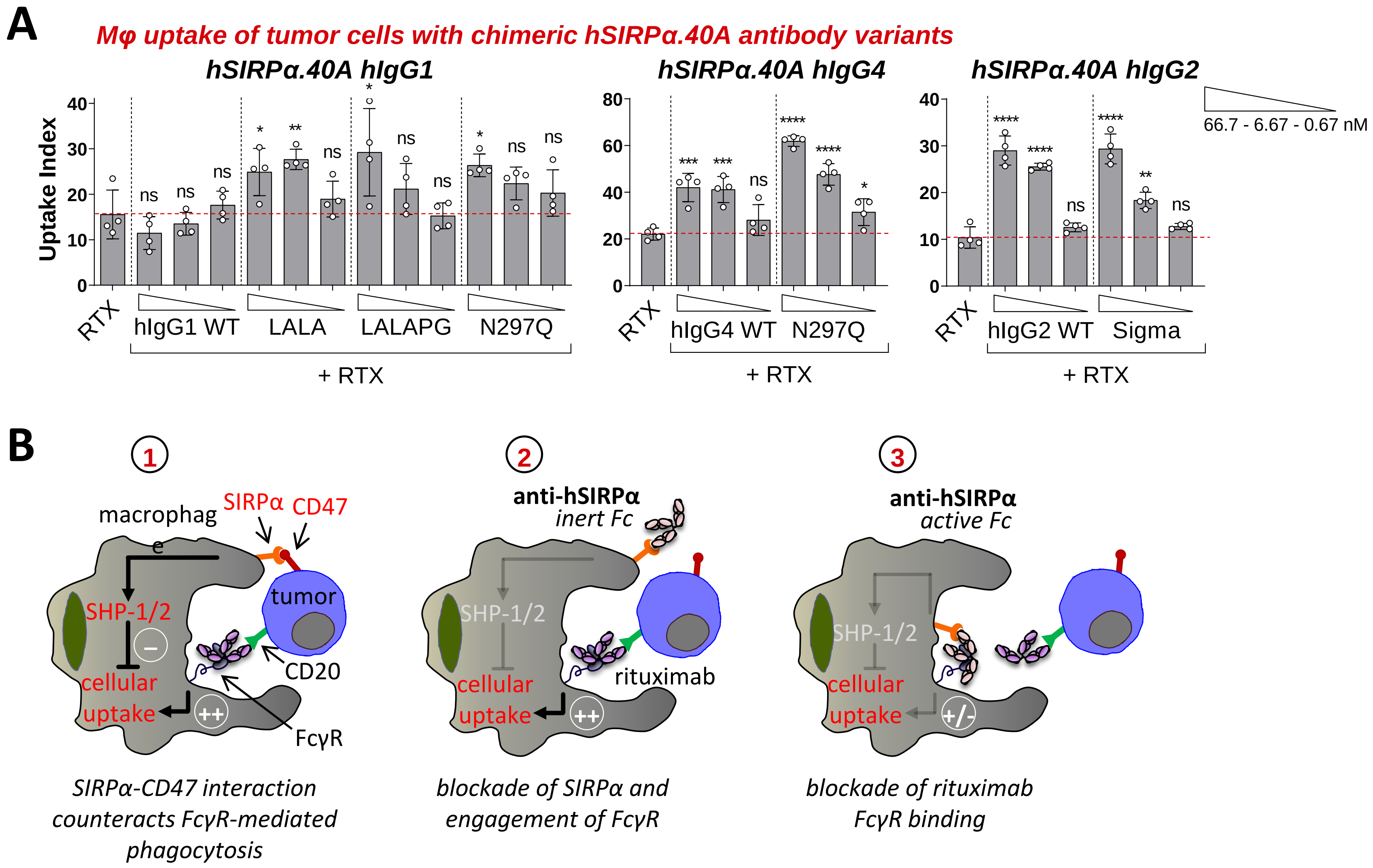

Additional file 7: Figure S4. Tumor cell uptake with anti-hSIRPα is most effective in absence of FcγR binding. (**A**) Chimeric hSIRPα.40A promotes optimal macrophage-mediated tumor cell uptake on a human IgG2 Fc backbone and also on engineered variants of a human IgG1, IgG2 or IgG4 Fc backbone. Abbreviations: LALA, L234A/L235A; LALAPG, L234A/L235A/P329G; Sigma, V234A/G237A/P238S/H268A/V309L/A330S/P331S. (Mean ± SD; representative of *n* = 4 is shown). (**B**) Model illustrating the 'scorpion effect': simultaneous binding of SIRPα and FcγR by anti-hSIRPα antibodies on the same cell. Anti-tumor antibodies, as exemplified by rituximab, engage FcγRs present on the macrophage and provide a positive stimulus for tumor cell phagocytosis. Under physiological conditions, SIRPα-CD47 interactions between macrophages and tumor cells constrain phagocytosis of these self cells (1). Blockade of SIRPα-CD47 interactions with anti-hSIRPα antibodies boosts FcγR-mediated phagocytosis (2). If these anti-hSIRPα antibodies harbor an Fc region (i.e., human IgG1, IgG4) that is capable of binding FcγRs, it is possible that the antibody simultaneously binds its target via the antigen-binding sites in the Fab arms and an FcγR on the same cell. This 'cis' interaction by the anti-hSIRPα antibody may compete with anti-tumor antibodies for FcγR binding and thereby dampen FcγR-mediated phagocytosis (3).
