# Supplementary material for: Functional characterization of the selective pan-allele anti-SIRPα antibody ADU-1805 that blocks the SIRPα–CD47 innate immune checkpoint
Source: J Immunother Cancer. 2019 Dec 4;7:340. doi: 10.1186/s40425-019-0772-0 (PMC6894304; doi:10.1186/s40425-019-0772-0)
Supplement: Supplementary file 8 — Additional file 8: Figure S5. Chimeric hSIRPα.40A does not impair platelet function. [file 40425_2019_772_MOESM8_ESM.pdf]

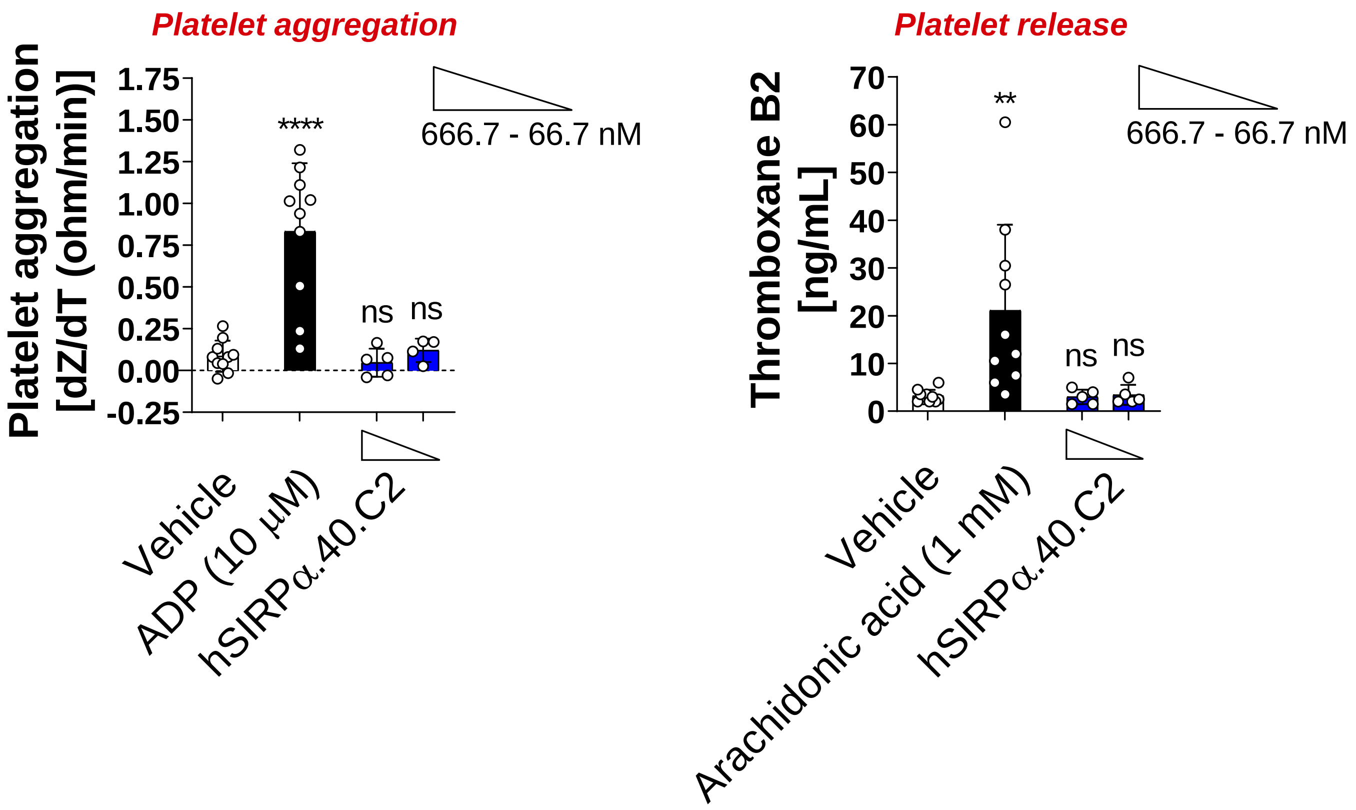

Additional file 8: Figure S5. Chimeric hSIRPα.40A does not impair platelet function.

Chimeric hSIRPα.40A (hSIRPα.40.C2) does not induce platelet aggregation or activation in human whole blood. Adenosine 5′-diphosphate sodium salt (ADP) and arachidonic acid serve as positive controls for the induction of platelet aggregation and platelet activation, respectively. Vehicle refers to the antibody formulation buffer: 10 mM L-Histidine pH 5.5 containing 0.1M sodium chloride. (Mean ± SD; at least $n = 4$ donors were tested per treatment). Data were analyzed by unpaired two-sided Student's t-test. * indicate statistical differences compared to the vehicle control group: **$p < 0.01$, ****$p < 0.0001$; ns, not significant.
