# Supplementary material for: Functional characterization of the selective pan-allele anti-SIRPα antibody ADU-1805 that blocks the SIRPα–CD47 innate immune checkpoint
Source: J Immunother Cancer. 2019 Dec 4;7:340. doi: 10.1186/s40425-019-0772-0 (PMC6894304; doi:10.1186/s40425-019-0772-0)
Supplement: Supplementary file 9 — Additional file 9: Figure S6. Anti-hSIRPα has a more selective binding profile as compared to anti-CD47. [file 40425_2019_772_MOESM9_ESM.pdf]

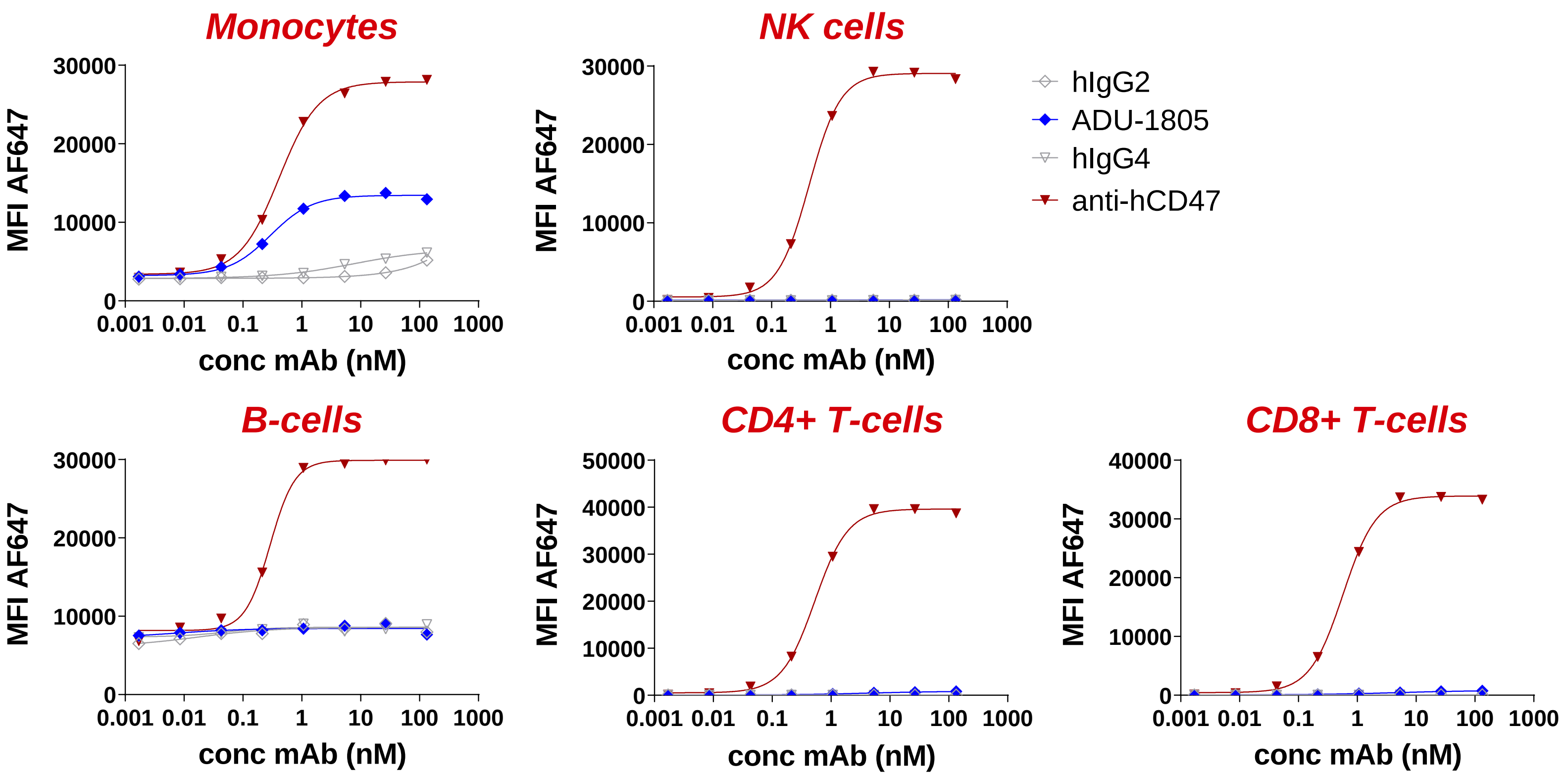

Additional file 9: Figure S6. Anti-hSIRPα has a more selective binding profile as compared to anti-CD47.

ADU-1805 binds SIRPα+ myeloid cells (i.e. monocytes) but, unlike anti-CD47, does not appreciably bind to human T-cells, B-cells and NK cells obtained from PBMCs. (Mean; representative of *n* = 3 donors is shown).
