# Supplementary material for: Functional characterization of the selective pan-allele anti-SIRPα antibody ADU-1805 that blocks the SIRPα–CD47 innate immune checkpoint
Source: J Immunother Cancer. 2019 Dec 4;7:340. doi: 10.1186/s40425-019-0772-0 (PMC6894304; doi:10.1186/s40425-019-0772-0)
Supplement: Supplementary file 10 — Additional file 10: Figure S7. Anti-hSIRPα does not impair CD4+ or CD8+ T-cell proliferation. [file 40425_2019_772_MOESM10_ESM.pdf]

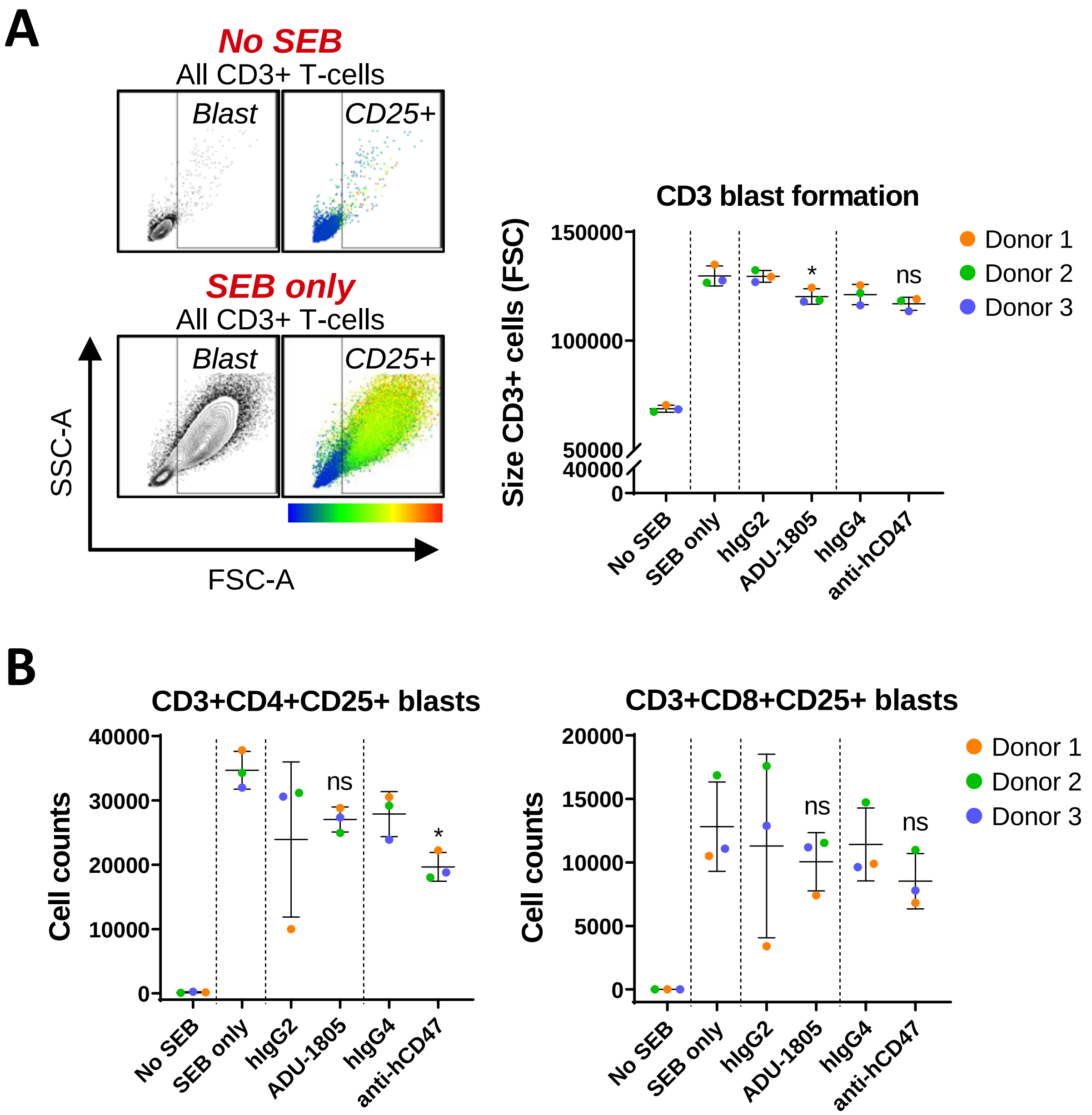

Additional file 10: Figure S7. Anti-hSIRPα does not impair CD4+ or CD8+ T-cell proliferation. (**A**) Illustration of CD3+ T-cell blast formation after 3 days treatment with SEB and indicated antibodies (used at 666.7 nM). Blast formation results in an increase in FSC-A and SSC-A. (**B**) Measurement of CD4+ and CD8+ T-cell activation. Anti-CD47 decreases CD4+ T-cell blast formation whereas ADU-1805 has no effect. (A, B: Mean; *n* = 3 donors are shown as a representative of *n* = 6 donors). Data were analyzed by unpaired two-sided Student's t-test. * indicate statistical differences compared to the respective isotype control group: *p < 0.05; ns, not significant.
