# Supplementary material for: Functional characterization of the selective pan-allele anti-SIRPα antibody ADU-1805 that blocks the SIRPα–CD47 innate immune checkpoint
Source: J Immunother Cancer. 2019 Dec 4;7:340. doi: 10.1186/s40425-019-0772-0 (PMC6894304; doi:10.1186/s40425-019-0772-0)
Supplement: Supplementary file 11 — Additional file 11: Figure S8. ADU-1805 is devoid of immune effector functions exemplified in complement and FcγR-dependent assays. [file 40425_2019_772_MOESM11_ESM.pdf]

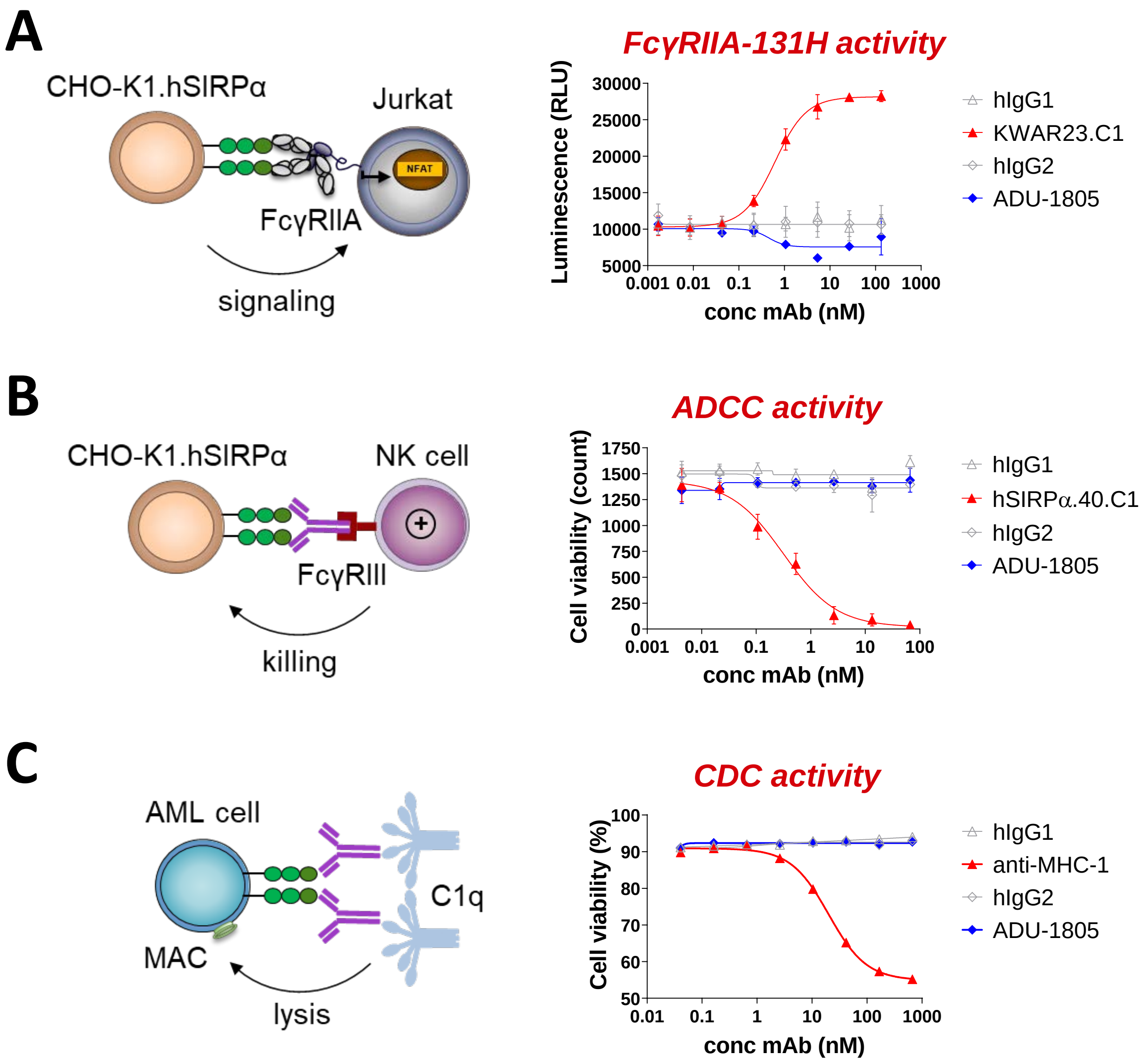

Additional file 11: Figure S8. ADU-1805 is devoid of immune effector functions exemplified in complement and FcγR-dependent assays.

(**A**) ADU-1805 does not engage FcγRIIA-131H. A human IgG1 variant of the chimeric KWAR23 antibody (KWAR23.C1; red line) represents a positive IgG1 antibody control that engages FcγRIIA-131H on Jurkat cells. (**B**) ADU-1805 does not trigger NK cell-mediated antibody-dependent cellular cytotoxicity (ADCC). A human IgG1 variant of the chimeric hSIRPα.40A antibody (hSIRPα.40.C1; red line) represents a positive IgG1 antibody control that induces ADCC. (**C**) ADU-1805 lacks complement-dependent cytotoxicity (CDC) activity. The anti-MHC-1 antibody (red line) represents a positive human IgG1 antibody control that induces CDC. MAC, membrane attack complex. (A, B, C: Mean ± SD; representative of *n* = 2 is shown).
