# Supplementary material for: Functional characterization of the selective pan-allele anti-SIRPα antibody ADU-1805 that blocks the SIRPα–CD47 innate immune checkpoint
Source: J Immunother Cancer. 2019 Dec 4;7:340. doi: 10.1186/s40425-019-0772-0 (PMC6894304; doi:10.1186/s40425-019-0772-0)
Supplement: Supplementary file 12 — Additional file 12: Figure S9. ADU-1805 does not induce cytokine release in human whole blood. [file 40425_2019_772_MOESM12_ESM.pdf]

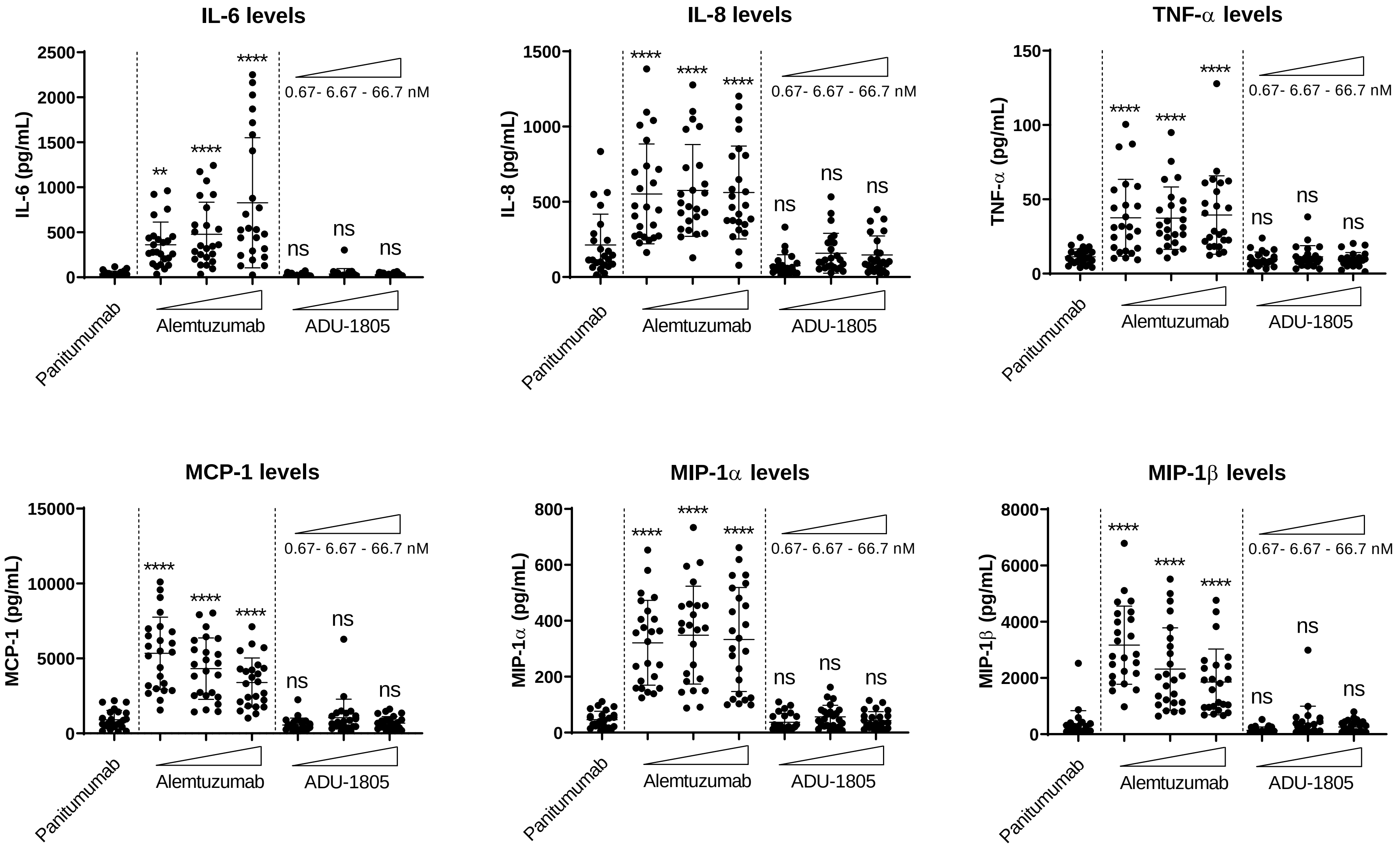

Additional file 12: Figure S9. ADU-1805 does not induce cytokine release in human whole blood.

Cytokine release was assessed in 24 human donors (each dot represents a single donor). Cytokine concentrations are obtained from 4x diluted supernatants that were taken after 16-24h. Results are shown for the following cytokines: IL-6, IL-8, TNF-α, MCP-1, MIP-1α, and MIP-1β. ADU-1805 and the positive control alemtuzumab (anti-CD52) were tested in a concentration range (0.67-6.67-66.7 nM); the negative control panitumumab (anti-EGFR) was tested at the highest dose only (66.7 nM). (Mean ± SD; *n* = 24 donors). Data were analyzed by one-way ANOVA with multiple comparison correction. * indicate statistical differences compared to the panitumumab control group: *p < 0.05, **p < 0.01, ***p < 0.001, ****p < 0.0001; ns, not significant.
