# Supplementary material for: Functional characterization of the selective pan-allele anti-SIRPα antibody ADU-1805 that blocks the SIRPα–CD47 innate immune checkpoint
Source: J Immunother Cancer. 2019 Dec 4;7:340. doi: 10.1186/s40425-019-0772-0 (PMC6894304; doi:10.1186/s40425-019-0772-0)
Supplement: Supplementary file 13 — Additional file 13: Figure S10. Cross-reactivity of ADU-1805 to cynomolgus monkey SIRPα. [file 40425_2019_772_MOESM13_ESM.pdf]

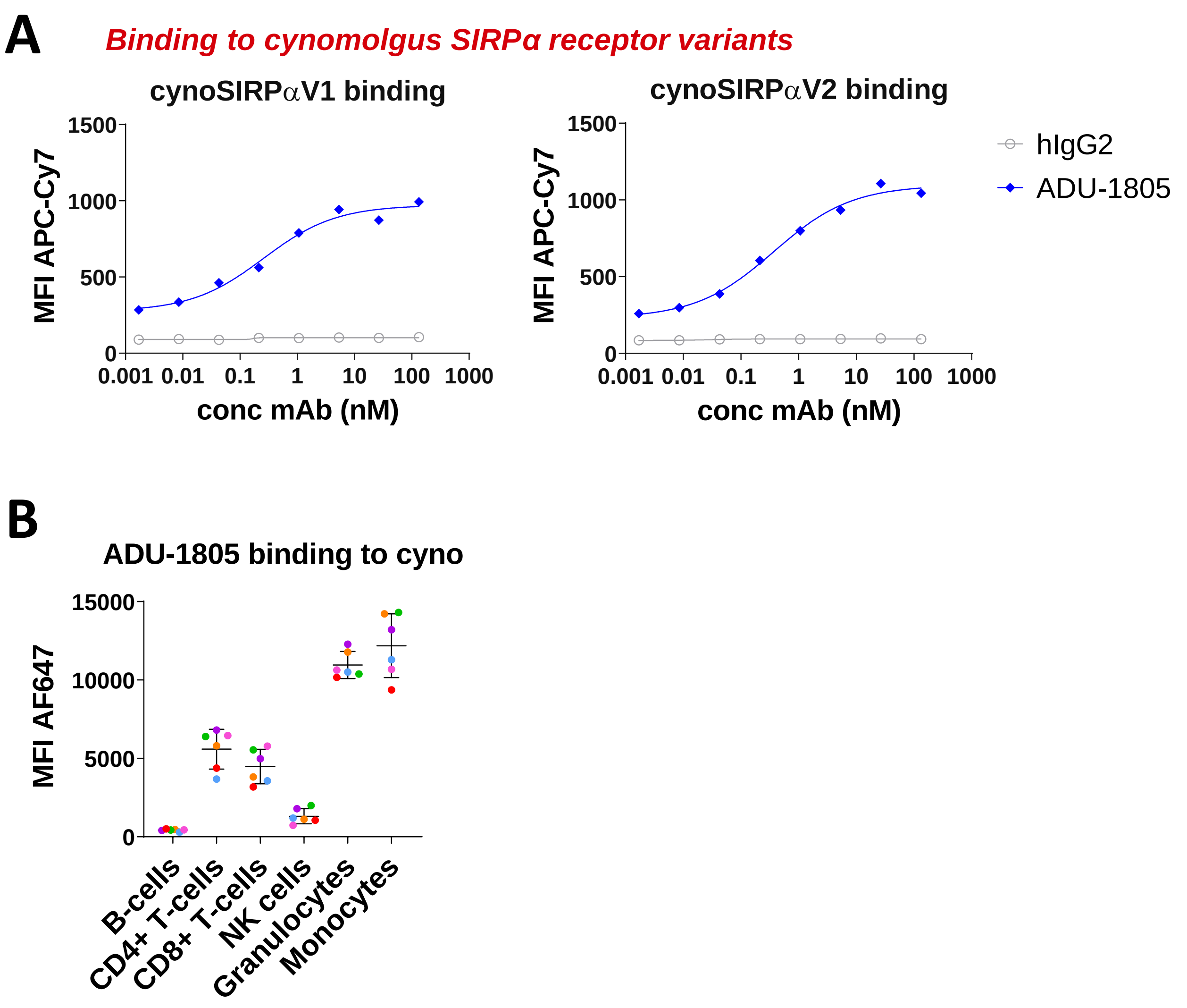

Additional file 13: Figure S10. Cross-reactivity of ADU-1805 to cynomolgus monkey SIRPα. (**A**) ADU-1805 binds to cynomolgus SIRPαV1 (NM_001284750.1) and SIRPαV2 (XP_015313155.1) expressed in CHO-K1 cells. (Mean; representative of *n* = 2 is shown). (**B**) Assessment of AF647-labeled ADU-1805 (used at 66.7 nM) binding to cynomolgus leukocytes present in erythrocyte-depleted whole blood (*n* = 6 animals). Antibody labeling of ADU-1805 was performed with Alexa Fluor 647 NHS Ester (Thermo Fisher Scientific) according to the manufacturer's instructions.
